# Smart patches for healthcare industry: a review of emerging technologies, challenges, and developmental opportunities

**DOI:** 10.1186/s12938-025-01485-3

**Published:** 2026-01-15

**Authors:** K Umapathi, L Priya, Hady Habib Fayek

**Affiliations:** 1 Department of Biomedical Engineering, KIT-Kalaignarkarunanidhi Institute of Technology, Coimbatore, India; 2 Department of Electronics and Communication Engineering, Sri Eshwar College of Engineering, Coimbatore, India; 3 Energy and Renewable Energy Engineering Department, Egyptian Chinese University, Cairo, Egypt

**Keywords:** Smart patches, Five sense organs, Nano-materials, Artificial intelligence, Remote healthcare

## Abstract

**Background:**

Smart patch healthcare devices are emerging as a distinct user interface in decoding the bidirectional interaction of the five sense organs. Powered by recent advancements in nano-materials, and artificial intelligence predictions, smart patches could understand the immune response of the body by analysing the biofluids, microenvironment and analytes in the five sense organs. These eminent potentials in smart patches, inspired the necessity for a review. Thus, this review aims to bring in to the limelight the current progress in smart patch technologies, highlighting their functions, opportunities and challenges in healthcare applications.

**Methods:**

A comprehensive review of literature was conducted focusing on smart patches designed for skin, ocular, cochlear, oral, and nasal applications. Further, the review is structured emphasising details on materials used, fabrication methods adapted, sensing mechanisms employed, enabling technologies such as artificial intelligence and Internet of Things.

**Results:**

The review analysis revealed that smart patches play a multifaceted role in healthcare applications providing (i) continuous health monitoring, (ii) controlled drug delivery, (iii) supports tissue regeneration and (iv) enables modulation of nerve responses. Further, smart patch integration with Internet of Things (IoT) capabilities enables remote healthcare solutions which benefits both physician and patient communities equally. Despite these progresses, challenges remain in term of biocompatibility of the materials chosen, long-term use and stability of the patch, data security and large-scale manufacturing.

**Conclusion:**

Smart patches hold transformative potential in biomedical engineering by bridging biosensing, therapeutic, and digital healthcare domains. This article provides an in-depth review of the current advancements, identifying the existing challenges and emerging opportunities in the field of smart patch research, and thus could guide future research and development. With its broad scope, this review would act as a valuable resource for both researchers and healthcare innovators working towards next-generation biomedical devices.

**Graphical Abstract:**

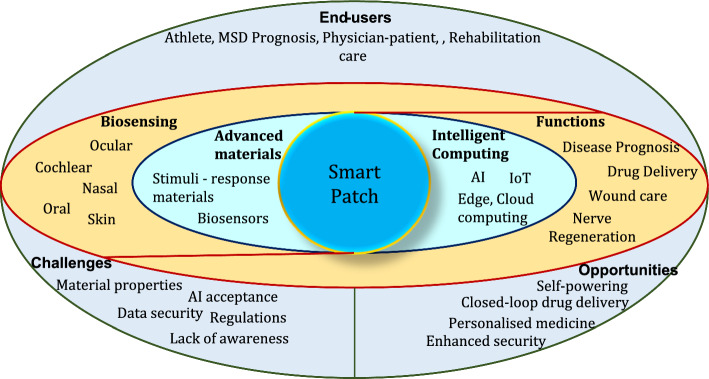

## Introduction

Smart patches have gained significant attention in recent times due to their potential real-time health monitoring in a non-invasive way. Further, smart patches facilitate enhanced healthcare solutions by enabling timely anomaly prevention by alerting patients and caretakers as well as provide precise drug delivery. The key component that drives developments in smart patches is wearable sensors. These sensors enable remote and real-time monitoring of patient health by physicians which has shifted intensive care units from hospitals to any convenient location of patient’s choice [1]. Such a device could benefit senior citizens, people affected by chronic conditions like diabetic, cardiovascular disease, sleep apnoea, seizures, people under rehabilitation therapy. In addition, these devices could be used for regular health monitoring of employees, sports team at reasonable cost.

The basic idea that drives development in smart patch technologies is well-routed in detecting/quantifying observable and unobservable changes in sense organs for non-invasive disease diagnosis. Examination of observable symptoms from the sense organs for disease diagnosis is a common practice in ancient medicine worldwide and is performed from time immemorial. Elementary science teaches that “Human sensory perceptions result from central nervous system’s response to the signals sent by the five sense organs”. However, studies reveal that sensory perceptions induce changes in the immune system in order to accommodate to the environment [2, 3]. Further, immune responses from the body are also expressed by these five sense organs in some form [4]. For instance, an immune response to respiratory illness may trigger running nose, dry cough, teared eyes, skin temperature rise, etc., and hence they act as preliminary sources of investigation by a physician for decades [5, 6]. Considering a chronic illness like diabetes, it may sometimes result in diabetic retinopathy which affects a person’s eyesight for their lifetime [7]. These examples support the fact that internal changes in the body induce changes in the fluids, analytes as well as the microenvironment in the sense organs. Thus, research interventions towards understanding and decoding this bidirectional interaction of the sense organs could create a paradigm shift in the way diseases are diagnosed [8]. Changes in analytes in the sensing microenvironment could be reliably be utilised for non-invasive sensing and early detection of diseases and thus, enabling better quality of life in numerous people. However, the primary limitation relies in the bio-comfort, biocompatibility and size of the sensors to be used with these organs. Thus, analyte sensing devices should be designed such that they do not hinder the normal functions of the organs at the same time do not induce harmful effects to the microenvironment. These stringent requirements of biocompatibility limit the clinical applicability of smart patch-based devices, though these devices have taken shape with the advancements in material science powered by nanotechnology advancements in communication technologies and artificial intelligence.

Non-invasive healthcare solutions are gaining significance inherent to their painless and patient-friendly nature. They offer numerous benefits, which include: (i) continuous health monitoring for various applications; (ii) prognosis; (iii) research and development of human machine interfaces. Further, recent day smart patch devices are equipped with multiple sensors and thus, enable multi-parameter monitoring. For instance, a smart patch with skin temperature sensors, skin conductance sensor and pulse wave sensor were fabricated and demonstrated to measure temperature and conductance with a sensitivity of 0.31 Ω/°C, 0.28 μV/0.02 μS and pulse wave with a response time of 70 mS. The patch is developed on a polyimide substrate with aluminium electrodes for temperature, conductance sensing and silver electrode with piezoelectric membrane for pulse wave sensing. It is fabricated following a time-controlled dipping along with polyimide wet etching process. The patch being capable of detecting multi-modal bio-signals is a potential candidate to quantify stress [9].

Smart patch devices have the potential to replace conventional invasive diagnostic procedure. These patches enable non-invasive solutions by making conformal contact with the epidermal surface, sensing biomarkers or bio-signals, and processing them to make reliable predictions. Such devices are equipped with nanoscale biorecognition elements capable of detecting even meagre amounts of biomarkers (even single molecule in some cases). Smart patches used for healthcare monitoring usually works on thermoelectric, pyroelectric, piezoresistive, triboelectric, capacitive, electrocatalytic or electrochemical sensing principles [10]. Materials that drive this change include: (i) substrates like polydimethylsiloxane (PDMS); polymethylmethacrylate (PMMA), polycarbonate (PC), polytetrafluorethylene (PTFE), polypropylene (PP), polyimide (PI), polyethylene terephthalate (PET), glass, silicon, cellulose paper; (ii) conductive materials like conductive polymers, carbon nanotubes, graphene materials, MXenes, transition metal complexes, and metallic nano-materials. In addition, fabrication techniques like 3D printing, PDMS printing, screen printing, UV laser writer, inkjet printing, laser ablation, wax printing, laser engraving, and photolithography enable simple, cost-effective realisation of such sensors [11].

Depending on their area of placement, smart patches can be classified as epidermal, dental, intracochlear, nasal or ocular [12]. Recently developed on-skin smart patches mimic the functions of natural human skin and such capabilities are made feasible with the introduction of flexible sensors that could measure temperature, humidity, strain, pressure, as well as sweat analytes [13]. Emergence of such biocompatible and smart patch is powered by the collective growth in medical science, material science, electronics, computing and communication technologies [14].

**Fig. 1:**
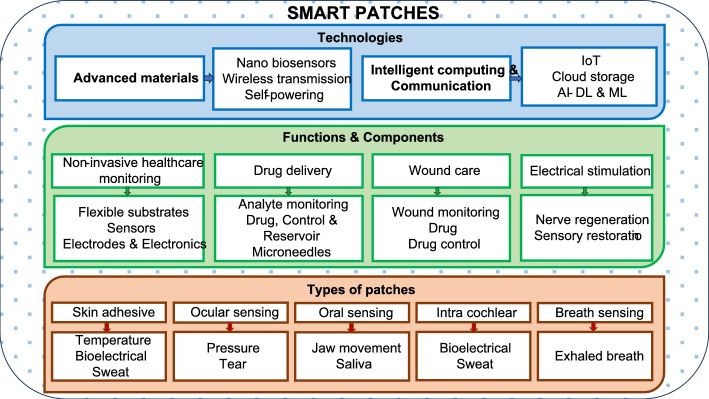
Types, functions and technologies that drive smart patches

Advancements in materials drive changes in (i) sensing; transducing, and transmitting signals; (ii) device powering and miniaturisation; (iii) flexible electronic realisations. Internet of Things (IoT) enabled efficient and fast communication of sensed data with the computing and storage resources in cloud. While artificial intelligence (AI) enables learned predictions for providing timely diagnosis and alerts. In spite of these benefits, there are numerous areas that demand improvements and challenges exist with respect to their development, commercialisation and user acceptance. Smart patches offer four different functionalities namely, non-invasive sensing, drug delivery, wound care and electrical stimulations. The types, functions and technologies that drive smart patch development are depicted in Fig. 1 below. Thus, this review is aimed to discuss the length and breadth of smart patches and their nuances. The objective of the review includes:

- To present a review of the literatures on smart patches that offer healthcare solution by sensing analytes and physiological parameters from (i) skin, (ii) ocular, (iii) oral cavity, (iv) inter-cochlear region or on ear, (v) breath or on nose.
- To elaborate the primary functions offered by smart patch devices namely, (i) non-invasive monitoring, (ii) drug delivery, (iii) wound care and (iv) electrical stimulations.
- To discuss the major technologies that drive advancements in smart patch developments which include: (i) material advancements powered by nanotechnology and (ii) intelligent computing, communication, and storage—powered by AI, IoT and cloud.
- To bring into the limelight the end-users benefited from smart patch technologies, challenges and opportunities that exist in smart patch research.

With this brief introduction, rest of the review is organised into four parts with Sect. “Part-I—smart sensing” discussing the types of smart sensing patches based on their attachment to body, followed by the functions offered by smart patches in Sect. “Functions of a smart patch device”. Sect. “Technologies that serve smart patches” discusses the technologies that drive research and development (R&D) in smart patches while the end-users benefitted, challenges and opportunities in (R&D) are elaborated in Sect. “End-users, challenges, opportunities”. Sect. “Materials and methods” presents the material and methods—which discusses the literature selection and filtration criteria adapted in structuring the manuscript.

## Part-I—smart sensing

This section discusses the material requirements, fabrication methods used for smart patch realisation along with representative examples. Smart patches can be broadly classified into two categories based on the nature of the parameter being monitored namely (i) physiological, and (ii) biochemical. However, different classifications exist. For instance, smart patches divided based on monitored parameter. As the primary function of a smart patch device is to offer non-invasive sensing of analytes for disease diagnosis, the smart patches are categorised under five major divisions based on their sensing microenvironment and considered for discussion in this section. With this basic introduction to smart patches and their categories, the detailed review of these categories is presented in the below topics.

### Skin adhesive patches

Skin attached biosensing methods are the oldest and conventional non-invasive measurement techniques that enabled ECG, EEG and EMG analysis. Recent day smart skin adhesive patches could measure (i) bio-electrical signals, (ii) skin temperature, (iii) stress–strain caused by motor actions, as well as (iv) analytes from sweat and thus, enable diagnosing numerous body conditions. The below discussions concentrate on various biomedical measurements from skin adhesive patch type devices. It starts with discussions on advancements in bio-electrical signal sensing techniques, followed by smart patches for skin temperature sensing and disease diagnosis from sweat.

*Bioelectrical signals sensing with smart patches.* Three of the most significant bio-electrical signals include ECG, EEG and EMG. Research and developments of smart patches capable of monitoring bio-electrical signals are little advanced when compared to patches that measure non-electrical parameters though there exist lot of opportunities for advancements [15]. For instance, Zio Patch^®^, is a smart ECG monitoring patch with cloud-based data storage and access enabling doctor’s on-desk diagnosis. This patch is approved by FDA for commercial use and can be worn continuously for 14 days reliably [16]. Recent day smart devices are capable of providing reliable ECG measurements even with single electrodes. For example, one electrode ECG recording, heart and lung sound recording by a patch type sensor with active noise cancellation by least mean square algorithm was developed and demonstrated in literature. The patch type sensor was able to transmit data to a tablet-PC via a USB. Such a patch is fabricated over a flexible substrate and had wireless data transmission capabilities [17]. In addition to patches, bio-signal sensing devices exist in the form of smart watches. Systematic review comparing the accuracy of ECH smart patches and photoplethysmography (PPG) smart watches revealed an equal performance while diagnosing atrial fibrillation (AF) [18]. Such patch type bio-signal sensing devices are used for monitoring heart failures, ST-elevation myocardial infarction (STEMI), as well as sleep apnoea in addition to AF detection [19].

Considering the measurement of EEG signals, patch type devices enabled the diagnosis of numerous cognitive health conditions [20]. For instance, a hybrid EEG–fNIR patch was developed and demonstrated to provide cognition and emotion detection. The patch was designed to be worn over the frontal brain region and could monitor cerebral haemodynamic response along with the EEG signals. Stroop task results confirmed that the patch could classify cognitive events and diagnose depressive disorder [21]. In addition, wearable smart patches were demonstrated to detect sleep apnoea with an accuracy of 88.5%. This smart patch was integrated with machine learning capabilities for predicting sleep scoring automatically. Such a smart patch system was realised by utilising two patches: (i) for measuring EEG and EOG from forehead and (ii) for measuring EMG from chin. The flexible patch was developed with a front side made from polytetrafluoroethylene (PTFE) and a highly stretchable and flexible nanomembrane electrodes for signal sensing. The captured signals are transmitted to a computing device such as mobile phone or PC through Bluetooth and the signals are processed by convolutional neural network (CNN) in real-time for sleep apnoea and sleep scoring detection [22].

Considering real-time monitoring of EMG signals enables determination of muscle-fatigue conditions. These measurement can be used for avoiding sports injuries during heavy exercises. Smart patches developed with two electrodes to measure EMG signals and equipped with ARMCortex-M4 processor was able to detect muscle fatigue by detecting shift in EMG frequency. It used an empirical mode decomposition algorithm for noise removal and root-mean-square value was used for computing the difference between real-time and off-line median frequencies [23]. Apart from diagnosing muscle fatigue, EMG smart patches are vastly used for capturing EMG signals to decode complex hand gestures, hand positions, joint angle predictions for training artificial intelligence models for enabling human machine interactions (HMI). Such AI algorithms are potential candidates for enabling prosthetics, assistive technologies that help people in rehabilitation setups and robot–human interactions [24].

**Fig. 2:**
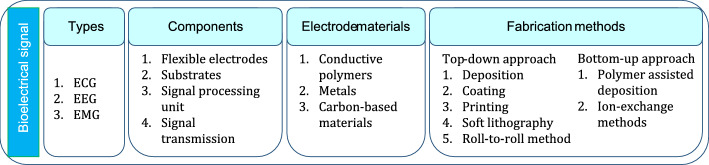
Types, components, materials and fabrication methods used for realisation of bio-signal sensing devices

*Flexible materials.* The emergence of flexible, biocompatible materials developed from natural polymers, integrated with nanoscale fabrication technologies drive developments in skin adhesive patches for sensing various bioelectric signals. These materials facilitate development of flexible electrodes and substrates that make conformal contact with the skin enabling reliable bio-signal measurement. Especially, bio-electrical signal transduction based patches that are capable of measuring EEG, EMG and ECG signals when designed with flexible electrode offer bio-comfort to the wearer. These flexible electrodes are either fabricated as active electrode array or passive electrode arrays and frequently use (i) metals, (ii) carbon-based materials or (iii) polymers as materials [25]. These flexible electrodes are fabricated by either top-down or bottom-up fabrication approaches. Top-down approaches deposit materials on substrate namely (i) physical evaporation deposition; (ii) Dip-Pen nanolithography technology; (iii) screen printing; (iv) inkjet printing; (v) soft lithography and (vi) roll-to-roll method while bottom-up approaches use; (i) polymer-assisted deposition; and (ii) ion-exchange methods [26]. For instance, a flexible surface EMG electrode is fabricated using tannic acid, poly vinyl alcohol and PEDOT:PSS. The flexible electrode (TPP) exhibited a stretchability of -200% and an adhesion of 0.58 N/cm. The electrode was able to ensure stable contact with skin for 5 days [27]. Amorphous indium gallium zinc oxide thin film transistors (aIGZO-TFTs) fabricated on flexible substrates could reliably measure bio-signals from human body [28]. The materials used and fabrication methods followed for the realisation of patch type bio-signal sensing devices are summarised in Fig. 2.

Next to bio-signal sensing, skin attached smart patch devices are used for measuring skin temperature. Temperature sensors play a significant role in diagnosing numerous health conditions like hydration status, wound condition, and general health condition of a person and thus carries significance.

*Skin temperature sensing smart patches.* Wearable smart patches are capable of providing prognostic solutions for biomedical applications. Human body temperature serves as a direct indication of an immune response [29]. Inherent to their application importance, developing fast responding, highly sensitive, stable, and reliable temperature sensors capable of measuring a wide range of temperatures is gaining research importance globally [9]. For example, a smart temperature sensing patch with fully integrated wireless transmission was developed and tested for evaluating feasibility and psychometrics related to continuous temperature monitoring. Study results favoured patients adaptability to flexible patch type sensors for continuous measurements. The temperature sensor which is 2.7 cm in length, used platinum electrodes and was fabricated by lithography technique [30]. Temperature changes could be reliable biomarkers for wound infection. For instance, a temperature sensing patch with integrated thermal heaters was fabricated by patterning 2-layer graphene using photolithography. Such a patch was demonstrated to offer continuous temperature sensing as well as thermal therapy for rapid wound healing [31]. Skin temperature measurement holds lot of biomedical significance in other than simple continuous skin temperature measurement. For instance, continuous temperature sensing obtained by placing 65 temperature sensing smart patches over the body was demonstrated to aid in monitoring circadian cycles and mitigating skin ulcer [32].

Designing accurate temperature sensing patches is a challenging task as skin temperature is often affected by environmental temperature changes and this directly reduces the accuracy of the device. However, the introduction of patterned fabricating methods based on printing/coating techniques and flexible substrates, enabled the realisation of flexible temperature sensing patches that perfect the function of electronic skin [33]. For example, temperature sensing patch with good sensitivity were fabricated by spray coating of reduced graphene oxide (rGO) over PDMS substrate using a polyimide mask. Such a temperature sensor was demonstrated to sense temperature in the range 25 to 70 °C [34]. In another experiment, smart temperature sensing patches were developed by inkjet printing of graphene ink over PDMS substrate followed by photonic sintering. This patch was equipped with wireless module for monitoring real-time temperature variations over a laptop and is capable of measuring temperatures in -20 °C to 50 °C [35].

**Fig. 3:**
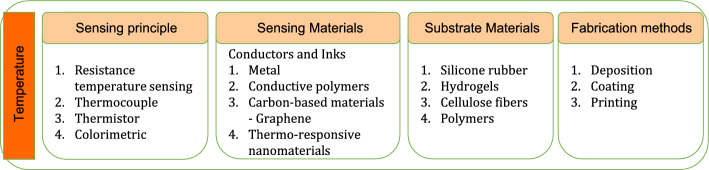
Sensing principle, materials and methods used for realisation of temperature sensors

*Materials and methods for temperature sensor fabrication.* Discovery and development of nano-materials remain as the backbone for smart patch technologies. Especially, flexible temperature sensors developed by depositing temperature sensitive materials on flexible substrates have the potential to serve as robotic skin in addition to functioning as biomedical temperature sensors [36]. These flexible temperature sensors were developed using temperature sensitive conductors and inks made from conductive polymers, metals or carbon-based materials like graphene, graphene oxide and reduced graphene oxides. Such flexible sensors are fabricated by printing, coating and deposition techniques and they work based on resistance temperature sensing, thermocouple and thermistor principles [37]. In addition to the sensing materials, even substrates play an important role as they provide the required biocompatibility. Materials like silicone rubber, hydrogels, cellulose fibres and polymers are frequently used as substrate materials for temperature sensing patches [38]. However, the emergence of nano-materials is creating a paradigm shift in the way sensors are realised. For instance, microgels developed from poly(N-isopropylacrylamide) (PNIPAM) decorated with gold nanoparticles (AuNPs) demonstrated colour change in response to temperature change. These thermo-responsive plasmonic microgels when embedding in stretchable hydrogel films were demonstrated to be potential candidates for smart calorimetric sensors and thermo-responsive actuators [39]. The sensing principle, materials and methods used for fabrication of temperature sensors are depicted in Fig. 3.

*Sweat sensing smart patches.* Sweat is osmotically related to blood and thus, contains metabolites that directly reflect the health status of an individual. However, the analyte concentration in sweat is lesser than in blood [40]. Sweat when used as an analysis medium could reveal metabolites (glucose, lactate), electrolytes, pH, drugs, and cortisol statuses which directly depict the hydration status, muscle function, and stress level in a person [41, 42]. These devices are broadly classified into two types namely, (i) electrochemical and (ii) optical (fluorescence or colorimetric) depending on the way output is produced. Colorimetric patches produce semi-quantitative results, whereas electrochemical patches are capable of producing exact numerical quantities [43]. An important requirement in sweat sensing patch design is their ability to stimulate and transport sweat to the sensing region. This is accompanied by two methods namely, (i) active (iontophoresis), and (ii) passive (exercise) methods. Iontophoresis involves sweat secretion by passing sweat-inducing substances like pilocarpine by applying electrical current while reverse iontophoresis involves extraction of ions from the skin by using a low current. Active stimulation methods are helpful in analysing sweat even under sedentary conditions [44].

**Fig. 4:**
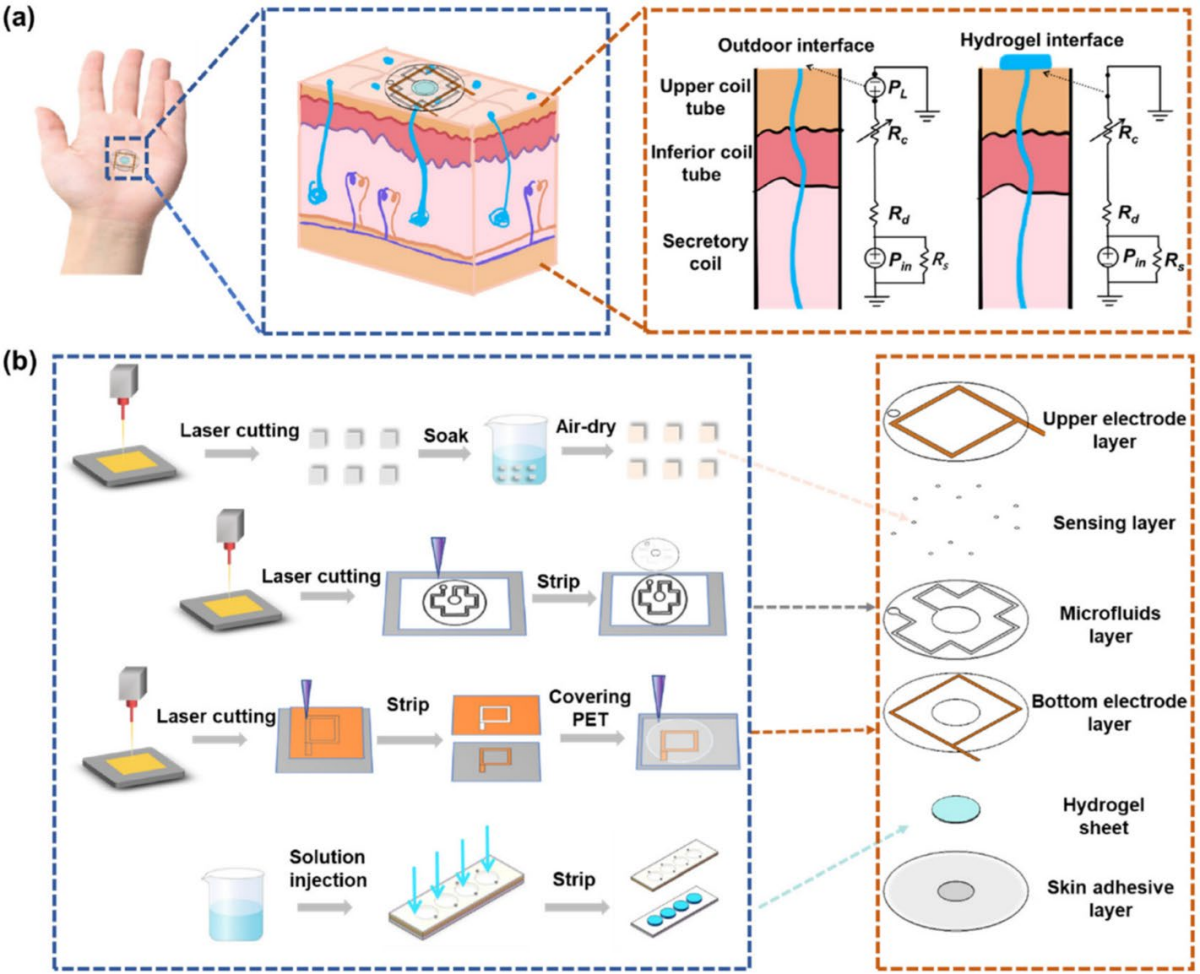
Schematic representation of a sweat sensing smart patch device. **a** Model depicting sweat detection using hydrogel interface, **b** steps involved in the fabrication of sweat sensing patch. Reproduced with permission from [49] under a Creative Commons Attribution (CC BY) license (https://creativecommons.org/licenses/by/4.0/)

Fully integrated smart patch sweat sensing systems were capable of measuring multiple analytes and physiological parameters together. For instance, a multiplexed flexible sensing array could quantify sodium, potassium, glucose, lactate in sweat along with skin temperature at the same time. The patch type sweat analysis sensor used ion-selective electrodes for sensing Na + and K + levels while lactate and glucose are sensed by using enzymatic amperometry sensors with Ag/AgCl two electrode system [45]. In another experiment, electrolytes (Na + , Cl-), glucose are quantified using a sweat sensing patch. The device is capable of making periodic predictions by iontophoretic sweat secretion and thus, could serve as a potential device for early cystic fibrosis diagnosis [46]. There are two types of sweat analysis systems based on whether analysis is done at the site of sweat secretion (in situ) or microfluidic chip is used to analyse sweat (ex situ). Conventional microfluidic systems use multiple valves and complex control modules to control fluid flow which is not suitable for miniaturised wearable smart patches and thus, novel fluid flow control methods are used in microfluidics for wearable devices. Three methods are frequently used for sweat sample collection namely, (i) capillary forces, (ii) evaporation driven pump and (iii) hydrogel osmotic pumps [40]. Sweat sensing smart patch capable of detecting pH, sodium ions, and uric acid with a hydrophilic and hydrophobic microfluidic synergy system for sweat collection was developed utilising laser engraving technique. Such a patch produced detection of analytes in a clinically relevant linear range of 4–8 (PH), 0–160 mM (sodium), and 5–160 μM (uric acid) [47]. Similarly, colorimetric fabric-based microfluidic system was designed based on hydrophilic–hydrophobic composite for sensing analytes from sweat. The patch could identify the presence of chloride ions and pH level in sweat by producing a colour-change reaction [48]. Further, Agarose, glycerol, hydrogel sheet integrated with microfluidic systems were demonstrated facilitate sweat collection in resting condition as well as analysis of multiple biomarkers simultaneously. A schematic representation of this smart sweat sensing patch is shown in Fig. 4. The sweat sensing patch could measure sweat rate, chloride ion and feature a colorimetric module to detect glucose level. RGB images of the colorimetric sensor are used for quantifying glucose levels [49].

**Fig. 5:**
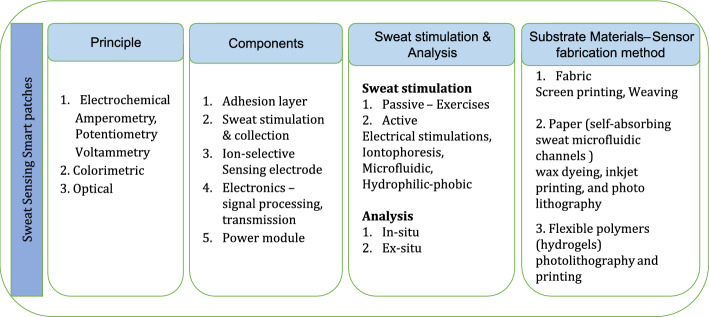
Principle, components and fabrication methods for sweat sensing patch fabrication

*Materials for sweat sensing.* Hydrogels are polymeric materials widely used as substrates that carry biosensors for skin attached smart patch sensors. Especially, sweat-based smart patches could achieve convenient and improved sampling efficiency with hydrogels that function as osmotic pumps for extraction and transportation of sweat for analysis. For instance, Agarose hydrogel with Dulbecco’s phosphate buffer saline (DPBS) solution was demonstrated to extract sweat continuously by using osmatic pumping mechanism and enabling measurement of L-lactate from sweat [50]. Similarly, calorimetric assays conducted using a hydrogel disc along with capillary action of paper channel revealed that, hydrogel discs are capable of extracting sweat by osmosis even when the wearer is at rest [51]. The types, components, sweat stimulation principles and sensor fabrication methods used for the realisation of sweat sensors are given in Fig. 5. Sweat sensing-based smart patches are fabricated from biocompatible materials like fabrics, polymers and sweat absorbing papers. Techniques like, photolithography are used for fabricating microfluidic systems in paper and polymeric substrates while screen printing and weaving are used for realising sweat sensing systems in fabrics.

*Limitations.* Sweat secretion methods such as iontophoresis may damage sweat glands which results in complete blocking of sympathetic transmissions from nervous systems. This results in raised threshold for nerve impulses [52].

### Smart patches in ocular microenvironment

Tear is the second (after sweat) vastly investigated biofluid for non-invasive healthcare monitoring. Tear carries traces of proteins, metabolites, electrolytes and lipids similar to blood resulting from plasma leakage between blood–tear barrier. This aids in predicting/identifying ocular diseases, diabetic and cancer [53]. Especially, ocular diseases such as conjunctivitis, dry eye disease and infections in eye can be effectively identified from tear [54]. For instance, fluorescent lysozyme assay of contact lenses worn by patients revealed that eye strain induces increased lysozyme concentration in tear fluid. However, in patients with dry eye disease (DED), the increase in lysozyme concentration is relatively low than in normal persons [55].

**Fig. 6:**
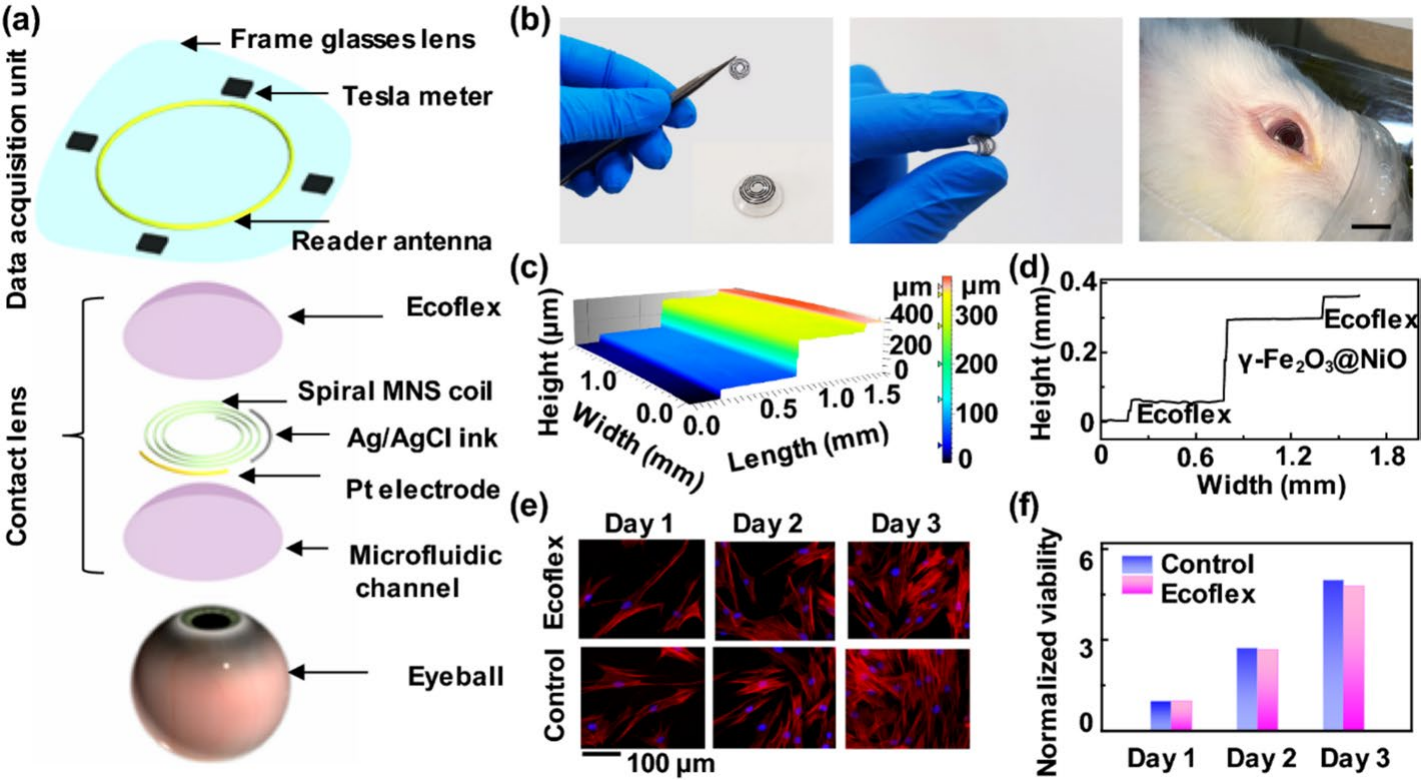
Schematic representation of smart contact lens: **a** the layers that constitute the smart contact lens; **b** digital images of the contact lens, normal state, bending state, tested on rabbit’s eye with 1 cm scale bar (from left to right) **c** 3D thickness diagram; **d** thickness ladder diagram; **e** cell culture fluorescence images of control and Ecoflex; **f** comparison of cell viability with control taken over 3 days. Reproduced with permission from [59] under a Creative Commons Attribution 4.0 International License, http://creativecommons.org/licenses/by/4.0/

Wearable smart tear sensors appear in three forms that include: (i) contact lens, (ii) flexible eye patch, and (iii) off-eye adhesive patches. Developments in smart contact lens technologies is powered by advances in biomaterials, biosensors, microfluidics, wireless communication, wireless power transfer as well as display circuits. And such smart contact lenses find application in drug delivery, glucose monitoring and disease biomarker detection in addition to diagnosing eye diseases [56]. For example, a flexible epidermal colorimetric patch type tear sensor was developed on PDMS substrate (crescent shaped for conformal contact with skin below eye) with microfluidic channels to sense vitamin C, H + , Ca2 + and proteins. A filter paper chip treated with chromogenic reagent acts as the colorimetric sensing agent developing colours in proportion to the biomarker concentration. A PDMS layer seals the patch top and a double-sided medical adhesive tape hold the patch sensor on one side and makes contact with skin in the other side [57]. Similarly, another colorimetric eye patch with faster response time (30 s) and capable of sensing pH, protein, ascorbic acid, and glucose from one drop (~ 20μL) of tears with AI and IoT supported monitoring was demonstrated in literature [58]. A multi-functional contact lens capable of monitoring glucose even at lower concentration in the order of 0.43 μmol was developed from inorganic magnetic oxide nanosheets (γ-Fe_2_O_3_@NiO). A schematic representation of the multi-functional smart contact lens is shown in Fig. 6. Such a lens was demonstrated to monitor eye pressure and movements with good accuracy [59].

Smart contact lenses integrated with wireless data transmission capabilities facilitate remote monitoring functions. For instance, an ocular contact lens capable of measuring glucose and ocular pressure from tear (by measuring resistance and capacitance variations) was developed and demonstrated to provide real-time monitoring capabilities. Wireless detection of glucose and ocular pressure were enabled by resistance–inductance–capacitance (RLC) circuit operating at radio frequency. The multi-functional sensor consists of an antenna and a field effect sensor fabricated using graphene–silver nanowire hybrid (Gr–AgNW), silicone elastomers. A graphene FET with graphene hybrid electrodes was fabricated and glucose sensing was made feasible by immobilising glucose oxidase on graphene channel by π–π stacking. Nine such FETs were fabricated over the lens with electrodes and interconnects passivated by epoxy and the square, sensing channel alone exposed for sensing glucose. Thus, AgCl formation from reaction of tear fluid with AgNW was prevented. Ocular pressure measurement was made feasible by an RLC circuit formed by placing silicone elastomers between two spirals of Gr–AgNW hybrid. Such a device was tested in vivo on a rabbit’s eye and in vitro using a bovine eyeball [60]. In addition, an optical glucose sensor was fabricated by printing photonic microstructures over phenylboronic acid functionalised glucose selective hydrogel. The sensor was capable of measuring glucose concentration in 0-50 nM range by corelating the first-order diffraction power with glucose concentration. The sensor was integrated with commercial contact lens and CGM data are monitored by smartphone camera readouts [61]. Even plasmonic etalon nanostructures were used for fabricating smart contact lenses capable of sensing tear glucose. Such a plasmonic sensor chip was fabricated by soft nanolithography over a contact lens [62].

*Materials for tear sensing.* The most important factor to be considered while designing smart sensing patches for ocular microenvironment is to ensure their biocompatibility in order to enable safe, irritation free, comfortable use [63]. Advances in material science and the emergence of nano-materials play a significant role in the realisation of electronic contact lenses capable of functioning as glucose sensors, drug delivering lenses and detection of disease biomarkers [64]. Especially materials such as (methyl methacrylate) (PMMA), poly (ethylene terephthalate) (PET), poly (2-hydroxyethyl methacrylate) (PHEMA), polydimethylsiloxane (PDMS), silicone, and 2-meth acryloyloxyethyl phosphorylcholine (MPC) serve as soft flexible substrates for smart contact lenses [56]. Hydrogels keep the cornea hydrated and allow oxygen permeability while silicone polymers enable oxygen permeability and provide the mechanical strength to lenses. Further, nano-coatings were used for ensuring antimicrobial functions and antifouling [65]. Fabrication techniques like lithography allow precise patterning and realisation of contact lens based smart devices while 3D printing techniques enable efficient realisation of microfluidic channels [66]. In addition to material selection, lens dimensions were adjusted for improving long-term wearability [63].

**Fig. 7:**
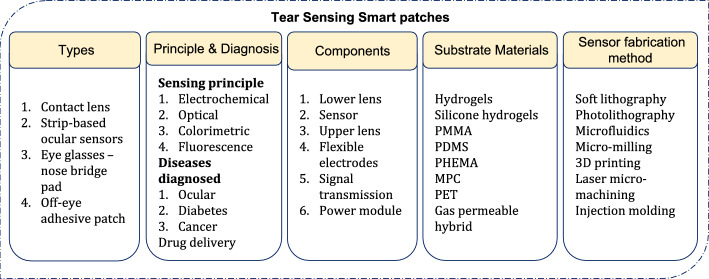
Types, principles, components, materials and methods used with ocular smart sensing

Nano-fabrication techniques and materials, like surface enhanced Raman scattering (SERs) contact lens material with a 3-layer structure which includes: (i) silk fibroin layer, (ii) silver nanowires (AgNWs) coated with 4-mercaptophenyl boronic acid (MPBA), and (iii) protective film was developed and demonstrated to sense glucose in tear in the range of 500 nM – 1 nM [67]. The types, principles, components, materials and methods used with ocular sensing patches are presented in Fig. 7 below.

## Limitations

Understanding the negative impact created by certain materials and technologies while designing sensors aid in saving time with design and testing and increase acceptability of the device. There are certain known impacts created by smart devices in the ocular microenvironment. First, the use of contact lens sensors with wireless transmission capabilities resulted in inducing physiological irregularities in eye. Specifically, oxygen transmission to cornea got reduced and interocular pressure got altered. These effects caused discomfort for wearer as well as resulted in challenging clinical interpretations and thus need to be addressed before realising a device [12]. Second is tear sampling method has direct impact on its constituents. Physiological stimulations alter the constituents of tear [53]. Thirdly, the use of opaque electronic sensing elements in the lens often obstructs vision. The use of plastic substrates is unsafe and result in low oxygen permeability. Further, presently available contact lens sensors enable monitoring single analytes. Advancements and multi-material integration with contact lenses is needed for sensing multiple biomarkers at the same instant [63]. Thus, proper material selection, precise design and adequate material testing are highly recommended while designing sensors for ocular use.

### Smart-sensing patches in the oral cavity

**Fig. 8:**
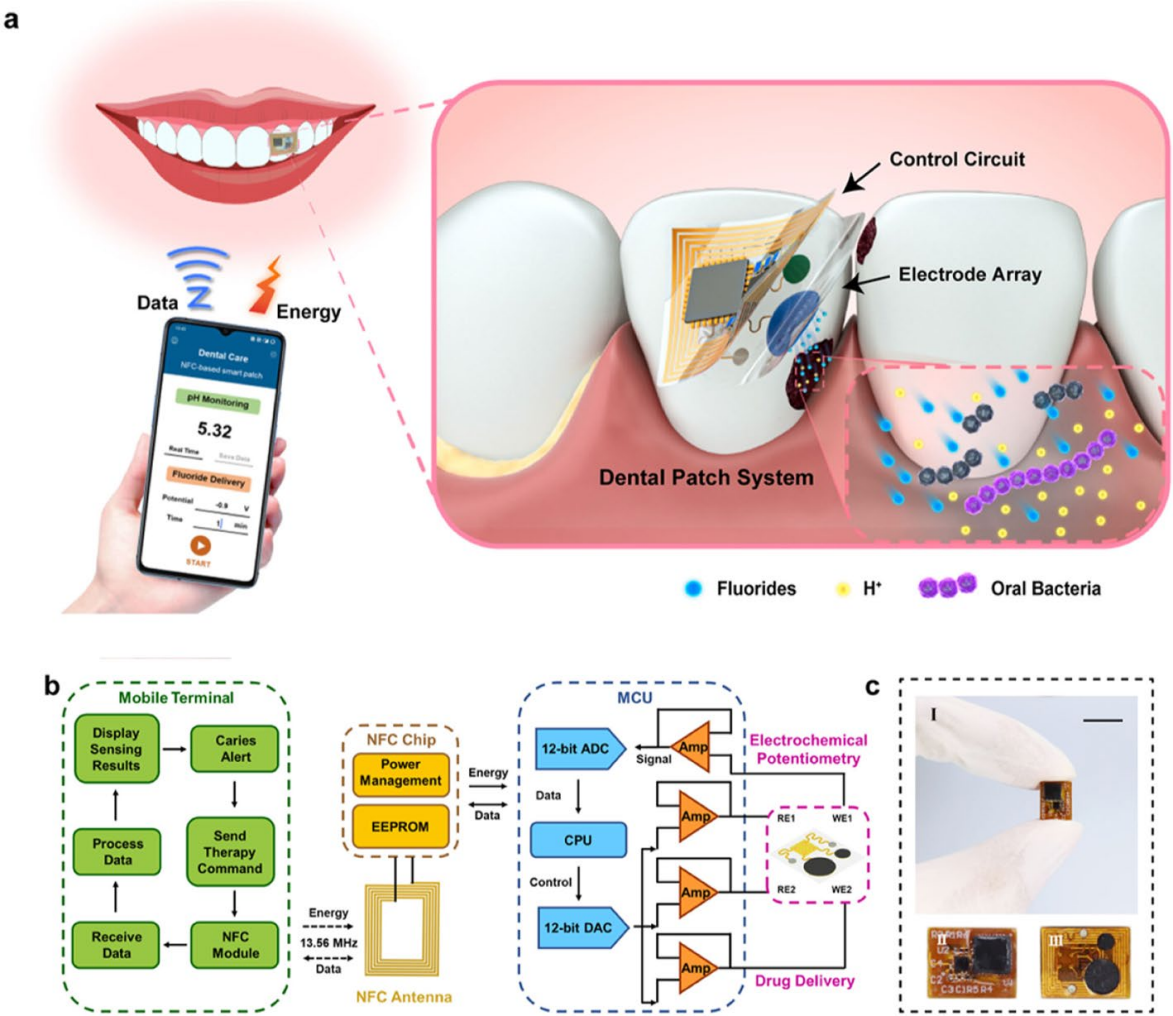
**a** Schematic concept of a smart dental patch; **b** workflow diagram of the processing and control circuitry; **c** digital image of the fabricated patch. (I) scale bar 1 cm, (II) front side electronic control circuit, (III) electrodes at the rear side. Reproduced from [73] under the terms of under a Creative Commons Attribution 4.0 International License (https://creativecommons.org/licenses/by/4.0/)

Oral cavity is capable of adapting easily to foreign substances and thus offers a comfortable sensing environment for hosting wearable sensors [68]. Although dental patches serve numerous functions, monitoring dental caries, dental implants and orthodontic treatment effects remain their major functions. In addition, salivary analytes can be monitored by dental patch sensors [69]. Saliva sensing for providing non-invasive healthcare solutions is gaining interest inherent to its stress free sampling and easy availability. Salivary biomarkers can be categorised into three major groups namely: (i) proteomic, (ii) genetic and (iii) metabolic biomarkers. These biomarkers enable detection of infectious diseases, neurological disorders, genetically inherited diseases, behavioural disorders, as well as metabolic variations in glucose, lipid [70]. Biomarkers like cortisol, chromogranin A, immunoglobulin A and neurotrophic factor derived from brain, α-amylase present in saliva could be effectively used for studying and diagnosing acute and/or chronic stressors. Biomarkers present in saliva could be sensed by a number of techniques like, surface plasmon resonance sensing, enzyme-linked immunosorbent assays, colorimetric assays, molecular imprinted polymer sensors, aptamers integrated electrochemiluminescence biosensing and so on [71]. A flexible and permeable facemask is designed and demonstrated to measure cortisol from artificial saliva. Even very low concentrations of 10 pM are sensed by the mask thus, revealing its excellent sensitivity [72]. Saliva sensors appear in the form of smart dentures, braces and dental patches [71]. These sensors are integrated with wireless data transmission to enable smart remote monitoring capabilities. For example, a wearable dental patch capable of monitoring acidogenic bacteria, offering controlled fluoride treatment, was capable of transmitting data wirelessly to a smartphone. The wearable battery-free patch was developed adapting a two-layer structure. The first layer fabricated over polyimide substrate was capable of wireless energy harvesting, data transmission and holds the control circuitry for drug delivery and sensing, consisting of microcontroller chip, resistors, capacitors, NFC chip and antenna. The second layer was fabricated over PDMS and holds electrochemical electrode array, sensing module and drug delivery module. Change in pH induced by acid synthesis of cariogenic bacteria was detected by electrochemical electrodes and corresponding signal was transmitted to smartphone. Drug release could be initiated by smart phone application. The electrode array was fabricated by screen printing of conductive copper ink over PDMS while control circuit and NFC module are designed for low power and accommodating limited space in oral cavity. NFC chip and antenna are designed for inductive coupling under a 13.56 MHz electromagnetic field [73]. An example of smart dental patch is depicted in Fig. 8 below.

In addition to disease diagnosis and monitoring, analyte concentration detection for quantifying drug concentrations is reliably made from saliva sensing. And thus, effectiveness of sensing methods in quantifying drug concentrations were studied in literature. For example, concentration of paracetamol in artificial saliva as measured by colorimetric and electrochemical sensors were quantified with MediMeter app and results support the efficacy of electrochemical sensors [74]. The introduction of nano-materials based biosensors acts as the driving force in realisation of oral cavity-based smart healthcare monitoring patches [75]. A wearable graphite bio-tooth sensor with wireless capabilities was developed and demonstrated to monitor tooth fracture by quantifying surface reflectance of tooth. In addition, the sensor is able to monitor salivary biomarkers for quantifying infections [76].

### Materials for saliva sensing

**Fig. 9:**
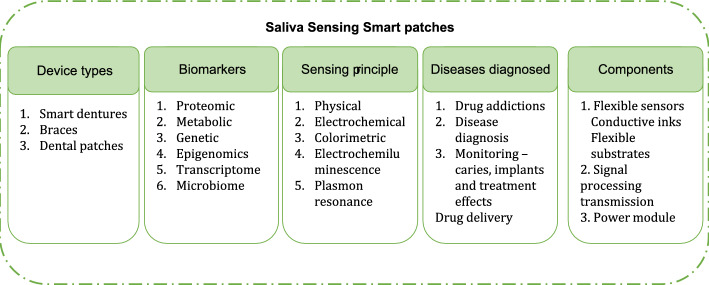
Types, biomarkers, sensing principles, diagnosis, components in an oral patch device

Oral cavity, saliva, carry numerous analytes and thus, facilitate detection of numerous biomarkers. However, substances in direct contact with saliva will be carried to the digestive system and hence spreads across the whole body. This necessitates use of inert and biocompatible materials for fabricating sensors when used with oral cavity. Graphene, graphene-based nanocomposites and carbon nanotubes are frequently used for fabricating salivary biosensors due to their biocompatibility [77]. Similarly, natural polymers like chitosan, hydrogel with enzymes, nanomaterial functionalised bio-polymers with colorimetric sensing are vastly used for fabricating oral sensors [78]. Further, smart sensors designed for the oral cavity are frequently made as small as possible in order to inhibit salivary flow rate [79]. Similarly, sensors were fabricated in the form of dental tattoos in order to make conformal contact with the tooth and provide reliable analyte measurements. Further, sensors in the form of splints, pacifiers, and mouth guards were designed for increasing wearer comfort [80]. However, these bulky devices often hurdle long-term wearability [81]. A list of device types, biomarkers present, sensing principle, diseases diagnosed and components that constitute an oral smart patch is shown in Fig. 9.

*Limitations.* Although the oral cavity is adaptable to foreign substances easily, it presents a least reliable place for hosting sensors because of the frequent physiological activities like eating, talking, breathing and the contaminants in oral cavity. Specifically, mechanical agitations resulting from eating could hurdle device adhesion and its long-term wearability. Similarly, constant saliva flow affects device adhesion capabilities [69]. Further, biofilm and pellicle formation over the oral sensors resulting from selective adsorption of macromolecules hurdles oral sensing effectiveness [68]. In addition, life of the oral wearable sensor greatly affects the usability of the device. Especially, in the case of implant and treatment monitoring devices, the life of the sensor should last at least for the life of the implant [69].

### Smart patches for intracochlear microenvironment

Smart materials serve intracochlear devices in two ways which include: (i) smart patch devices capable of monitoring various neuronal functions and electrochemical activities of ear and (ii) as sensors with hearing aids and implants.

**Fig. 10:**
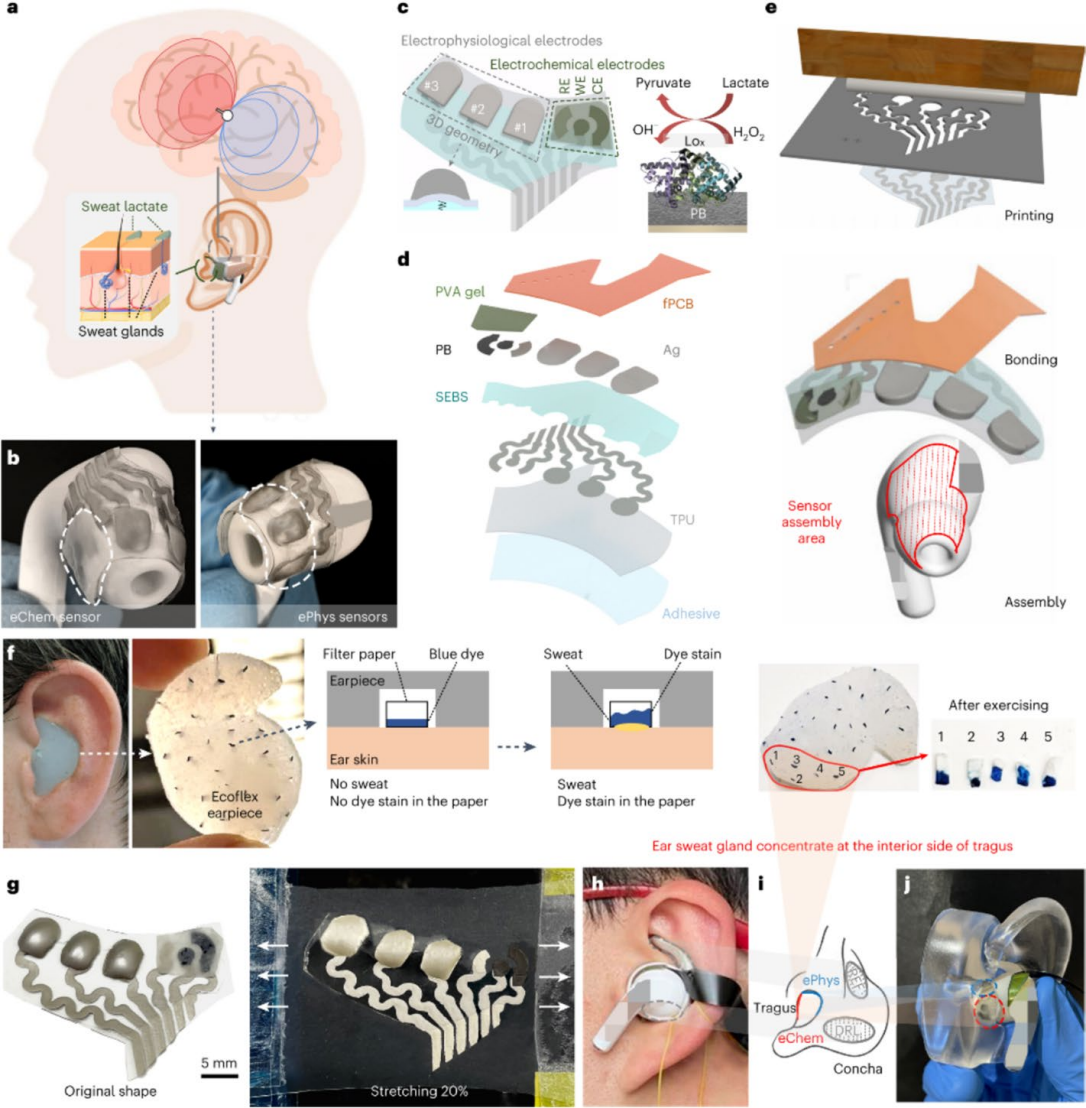
Smart ear patch device. **a** Schematic representation of sample in-ear sensing device; **b** representative image of fully integrated device; **c** overall layout of the sensing electrode array; **d** layers that constitute the in-ear smart sensing device; **e** sensor fabrication steps, printing, integrating electronics, assembly; **f** sweat mapping and colorimetric indication; **g** stretch resistance demonstration; **h** sensor in-use; **i** sensor geometry; **j** skin contact of sensors. Reproduced with permission from [85] under a Creative Commons Attribution 4.0 International License, http://creativecommons.org/licenses/by/4.0/

*Smart patches for monitoring.* Scalp caps or head bands are not appropriate for daily monitoring of EEG signals while patch type sensors attached behind-the-ear could reliably measure EEG, ECG, photoplethysmogram (PPG) as well as galvanic skin response (GSR) [82]. A graphene skin patch (GSP) fabricated on polyimide-Nomex fabric using laser-induced graphene was demonstrated to provide multi-modal signals proportional to external auditory canal in addition to delivering superior audio quality by utilising triboelectric nanogenerator (TENG), thermoacoustic (TA) and thermosensitive principles. Thus, enabling early warning, integrated health monitoring, and human machine interface (HMI) [83]. Further, an ear piece equipped with multiple sensors was demonstrated to measure neural, cardiac, speech and respiratory activity (both during sleep and awake) efficiently [84]. An in-ear smart device was developed using electrophysiological and electrochemical sensor arrays for monitoring brain states from ear sweat. A schematic of the ear patch system is shown in Fig. 10. The device was demonstrated to monitor brain states and lactate concentration from electroencephalography, electrooculography and electrodermal activity. Experimental results demonstrated that lactate level in ear sweat modulates with brain activity [85].

*Smart patches for drug delivery.* Cochlea is a challenging target for drug delivery; however, with recently developed materials and microfluidic patches, direct drug delivery to cochlea is made feasible. Such patch systems enable extended delivery of drugs and aid in restoring hearing loss resulting from auditory diseases [86]. Hydrogel- and nanoparticle-based drug delivery systems along with microneedles, micropumps, and cochlear implants enable treating inner ear diseases efficiently and aid in restoration of lost hearing [87].

*Smart sensors with implants.* Conventionally, intracochlear devices were used for treating hearing impairments and cochlear implants are the most successful neural prosthetic devices that aid in restoring hearing loss by direct electrical stimulation of auditory nerve. Acoustic signals of biomedical importance are of two types and they are: (i) external signals—received from environment and sent to the cochlea from where it is transferred to brain for interpretation; (ii) internal signals—resulting from functioning of internal organs, examples: heartbeat, pulsatile flow of blood. MEMS-based devices are used for the continuous and reliable interpretation of both these types of sound signals. These sensors are of four types which include: (i) piezoresistive or piezoelectric sensing element is embedded in an oscillatory cantilever—bending of cantilever induces voltages; (ii) sensing element is embedded in a diaphragm that vibrates with sound signals; (iii) integrating capacitive sensing mechanism to bending element; (iv) capacitive sensing-based diaphragm [88].

### Materials used in intracochlear microenvironment

Recent day smart materials when integrated with hearing aids and implants are capable of providing additional parameter measurements ranging from pressure, chemical environment assessment and so on. For instance, platinum-based stimulation electrode in cochlear implant was converted to an electrochemical sensor to study the chemical environment around the implant and the electrode status. The electrochemical sensor could reliably measure dissolved oxygen and hydrogen peroxide producing a linear and stable response, without disturbing the electrical stimulation function of the electrode [89]. In addition, experimental results revealed that ECoG recording could be reliably obtained from intracochlear implant electrodes [90]. Such sensor integrated implants are the need of the hour to provide, harmless and reliable solutions with implantable devices but such a technology is still in infancy [91]. With the development of nano-materials that respond to stimuli and exhibit multi-functional properties, such smart sensing capabilities both integrated with implants as well as in the form of standalone monitoring devices have become feasible [92].

Piezoceramic flexible patches (PFPs) are one such materials that respond to temperature changes and thus, could be used for real-time monitoring [93]. In another experiment, piezoelectric pressure sensor was fabricated by embedding polyvinyl fluoride (PVDF) film in PDMS by injection moulding and the capability of the sensor to measure sound pressure at the round window based on inputs given at external auditory canal (EAC) was studied. Results supported the PVDF sensor’s ability to detect sound pressure response to acoustic input given at EAC. Thus, by embedding PVDF sensor to conventional cochlear implant electrode, the device can induce stimulus as well as measure intracochlear pressure at the same time [94]. Advancements in implantable sensor technologies is revolutionising the real-time monitoring of biophysical and biochemical parameters by providing closed loop control and therapeutic solutions [95]. Though initially intended for sensing the health of implant, the integration of smart sensors with implants enabled a bidirectional interface in the intracochlear region.

### Smart patches for breath sensing

Analysing biomarkers in breath could reveal numerous health indicators related to respiratory conditions, and cardiovascular health. In addition, respiratory patterns, air flow, temperature and humidity can be found from breath analysis [96] [97]. Exhaled breath includes volatile organic compounds (VOCs), semi-volatile organic compounds (SVOCs), proteins, lipids, DNA, bacteria, and viruses which could be used for various diagnosis and health monitoring functions. Specifically, exhaled breath contains about 3500 VOCs resulting from metabolically excreted products and reach exhaled air through alveoli in lungs. Increase in concentration of nitric oxide (NO), carbon monoxide (CO), ammonia (NH3), hydrogen dioxide (H2 O2), sulphide (H2 S), ethanol, and acetone in breath indicate presence of diseases in patients [98]. Conventionally, mass spectrometry methods coupled with gas chromatography, selected-ion flow-tube (SIFT), and proton-transfer-reaction (PTR) are used for breath analysis.

#### Materials for breath sensing

**Fig. 11:**
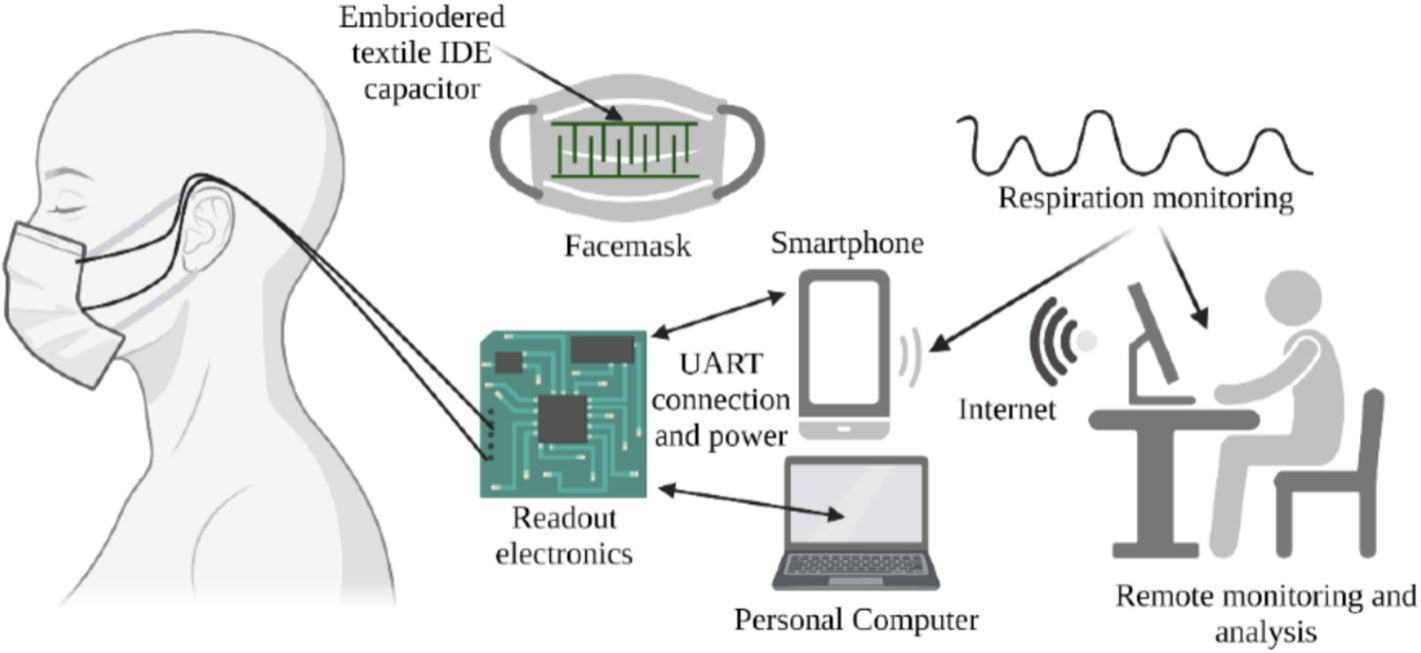
Schematic representation of respiration sensing smart face mask system. Reproduced with permission from [102] under a Creative Commons Attribution (CC BY) license (https://creativecommons.org/licenses/by/4.0/)

Recent developments use electrochemical sensors in the form of transistors with selectivity for a particular gas for gas analysis. For example, carbon nanotubes (CNTs) and 2D MXene are used for O2 and ammonia detection. Similarly, platinum, palladium, and gold NPs while coming in contact with ammonia change resistance which favours their use in gas sensing. Integrating individual gas sensing elements in array form enable sensing a wide variety of gases [99]. Elevated levels of O2 in breath result in hyperoxia while depletion leads to hypoxia. Similarly, increased CO2 leads to respiratory acidosis (confusion, lethargy, and in severe cases, respiratory failure) and depleted CO2 leads to respiratory alkalosis, (dizziness, light-headed ness, and, in severe cases, fainting). Hydrogel networks with embedded ions could be reliably used for O2 sensing while luminescent metal oxide semiconductors that use UV light are capable of sensing CO2. Even ammonia and hydrogen peroxide sensing carry significance in biomedical diagnosis [100]. In addition, pathogen detection from breath is feasible if characteristics of disease is known. Wearable breath sensors often take shape as either patches or as face masks [101]. For instance, a face mask type respiration sensor was demonstrated to monitor respiration status reliably. A schematic of this face mask type device is shown in Fig. 11. Such a device could benefit detection of various diseases conditions like cardiac arrest, asthma or sleep apnoea reliably [102].

**Fig. 12:**
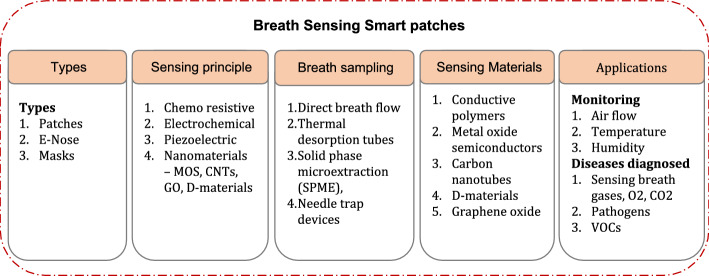
Types, principles, sampling methods and materials for breath sensing

Flexible substrates and printable conductive inks play a vital role in development of flexible respiratory sensing patch devices [103] [104]. A patch type respiratory sensor fabricated using a triboelectric film was demonstrated to measure respiration depth, frequency, respiration interval time and apnoea hypopnea index, peak expiratory flow and vital capacity with a sensitivity of 0.719 V/(m/s) under an air flow of 7 m/s speed [105]. Electrophysiological signals sensing patch was fabricated using a bi-phasic Ag-EGaIn composite printable ink and the sensing performance of the patch was demonstrated to be better in comparison with Ag/AgCl electrodes [106]. A mask type exhaled breath condensate analysis device was fabricated utilising tandem cooling strategy along with microfluidics, electrochemical sensors and wireless transmission. The device was demonstrated to assess EBC analytes continuously and in real-time depicting their ability to emerge as a potential multi-modal signal analysis system [107]. E-Nose techniques based on exhaled breath analysis and artificial intelligence are capable of detecting lung cancer with 71% to 96% sensitivity and 33 to 100% specificity [108–110]. TENG-based respiratory sensor data were integrated with signal processing unit and wireless transmission capabilities which enables remote monitoring of respiratory status [105]. The types of breath sensing devices, sensing principle, breath sampling methods used commonly, materials for breath sensing patches are depicted in Fig. 12 below.

Comparing the materials, fabrication methods and limitation of smart patches used across various sensing micro-environments the following conclusions are derived. Smart patches are interfaced with the sense organs and thus, three primary requirements (for material) arise for their reliable use. They are (i) biocompatibility; (ii) the device should not affect the normal functions of the microenvironment; (iii) adhesion and long-term wearability. Biocompatibility requirement is often met with the selection of proper materials while device miniaturisation and fabrication techniques are tuned for ensuring proper device functions without affecting the normal functions of the organs. However, these requirement differ across the microenvironment. Specifically, ocular patches should be transparent to enable proper vision and are usually made thin for comfortable long-term wearability. Transparent, soft flexible hydrogels and silicone are used as substrates for fabricating smart contact lenses. Further, the use of electronics with lens type ocular sensors for wireless transmission functions is highly challenging. Considering sweat-based patches, sweat sampling becomes a significant task. Especially in case of patients in ICUs, iontophoresis-based sweat sampling techniques are employed for obtaining sample. As sweat absorption becomes a primary requirement, paper and fabric are vastly used as substrates for sweat sensor fabrications. In the case of oral patches, salivary flow, mechanical agitations are hurdles against reliable placement of devices, while bio-fouling and biofilm formations are hurdles to long-term wearability. These requirements necessitated either extreme miniaturisation for conformal contact without being affected by saliva flow or use of patches with dental braces, mouth guards, pacifiers for long-term wearability and frequent dis-infection to avoid oral contamination. Graphene and natural polymeric materials are often used for fabricating saliva sensors. Considering breath sensing, use of patch type devices are highly limited and thus, mask, e-Noses type devices are often used for breath analyte sensing. Device miniaturisation is challenging in case of breath sensing devices. Metal oxide semiconductors, graphene compounds are vastly investigated for use with breath sensing devices. Developments in intracochlear implants and devices were quite successful to the extent of providing hearing aid to patients. And thus, researches were carried out towards understanding the health of implants and devices in intracochlear microenvironment. Considering fabrication strategies, almost all types of smart patches could be fabricated by following lithography or printing techniques. Lithography techniques are costlier however, could achieve precise patterning of sensor while 3D printing techniques could achieve cost-effective solutions for 3D realisations. Screen printing, inkjet printing of conductive materials are even more economical, however careful layer-by-layer fabrication should be adopted for realising the whole sensor unit.

## Functions of a smart patch device

The previous section discussed the material requirements, fabrication techniques used for realisation of smart patch devices with representative examples while this section reveals the functions offered by smart patches.

### Non-invasive multi-functional monitoring

**Fig. 13:**
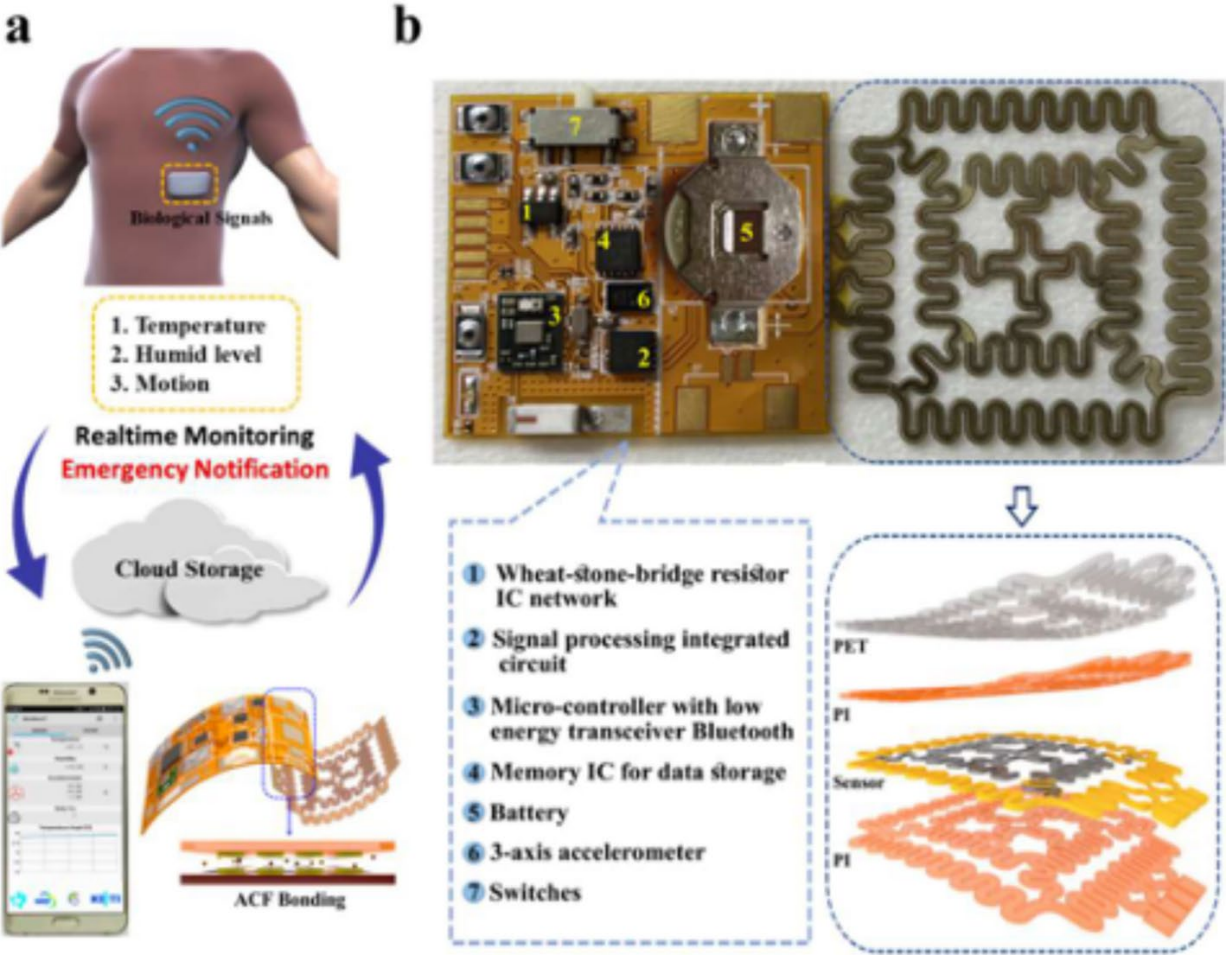
A non-invasive healthcare monitoring smart patch. **a** Schematic concept of a multi-functional smart patch device; **b** digital image of the sensing and processing units. Fabricated serpentine temperature–humidity sensor, is indicated by an arrow below (**b**) and its functions are depicted left to it. Reproduced from [113] under the terms of under the terms of under a Creative Commons Attribution 4.0 International License (https://creativecommons.org/licenses/by/4.0/)

Recent day smart patch devices incorporate multiple functions in a single patch in contrast to the initially developed single parameter monitoring patches. Interestingly, these patches could incorporate functions of bulky traditional medical devices in thin flexible patches by making use of nano-materials and flexible electronics technologies [111]. For instance, a multi-functional patch devices with inherent calibration tuning capabilities were developed. The patch capable of monitoring ECG signals and sweat glucose was integrated with temperature and pH sensors for calibrating glucose sensor with pH and temperature changes. The device was fabricated adapting microelectromechanical system (MEMS) fabrication steps for realising device layers and functionalisation of electrochemical sensors by drop casting and electroplating techniques. Glucose sensor was fabricated by drop casting rGO over Au electrodes followed by electroplating PtNPs and immobilising GOx. Finally, the sensor was coated with naflon. While pH sensor was fabricated by electroplating PANI over Au electrodes. ECG electrodes and temperature sensor were realised by patterning and lift-off processes [112]. Similarly, a kirigami-serpentine shaped temperature sensor is fabricated by depositing platinum (Pt) thin film on patterned, PI coated, glass substrate. Reactive ion-etching is performed to remove photo resist after Pt deposition. In addition, an interdigitated serpentine shaped humidity sensor capable of measuring humidity from capacitance changes is fabricated following the same procedure. Gold electrodes are attached to pick up variations from the sensors. The heterogeneous sensor surface is coated with polyethylene-terephthalate (PET) film and bottom PI layer over glass substrate is peeled off manually using a tweezer. The flexible patch type sensor was interfaced with a microcontroller with BLE module and demonstrated to sense and transmit real-time temperature and humidity variations effectively [113]. Further, wearable microneedle patch capable of monitoring glucose and lactate (in the range 0.3–30 mM) continuously from dermal ISF was developed and demonstrated to detect pre-diabetes, metabolic syndrome or sepsis early. The microneedles used in ELSAH system are 0.5 mm and thus are painless. The microneedle patch could measure glucose, lactate and transmit the measured values to smart phone wirelessly [114]. Smart patch devices integrated with AI techniques for cardiovascular health monitoring are increasing. These devices use photoplethysmography, pulse pressure, bioimpedance analysis, electrocardiogram, ultrasonography and seismocardiography/ballistocardiography principles [115]. For instance, stress cardiomyopathy which is conventionally diagnosed only after a physical damage to heart muscles, could now be easily prognosed by identifying biomarkers like cortisol, dopamine, epinephrine, α-amylase, catecholamine, norepinephrine, and serotonin [116]. In addition, smart patch devices hold immense potentials to offer personalised medicine by their abilities (i) to collect an individual’s health status at molecular level and (ii) predictive analysis [117]. These patch type devices offer non-invasive monitoring and early disease predictions. An example of non-invasive healthcare monitoring smart patch is presented in Fig. 13 below.

### Drug delivery

**Fig. 14:**
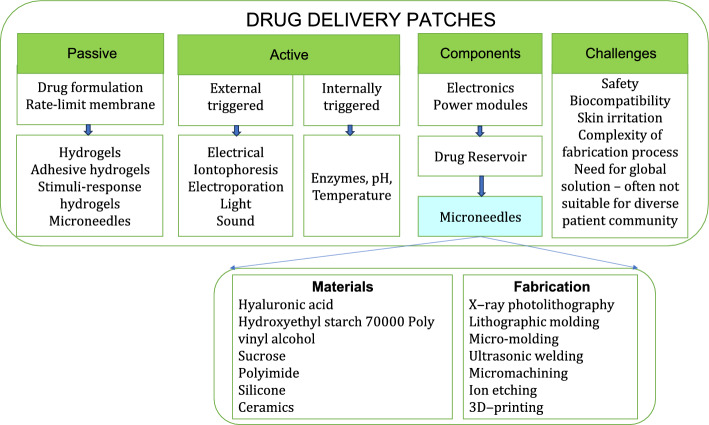
Drug delivery patches and their components

Next to biosensing, smart patches are adequately investigated for drug delivery applications. Smart drug delivery systems ensure patient compliance, drug availability at the same time avoids gastrointestinal degradation. There are two types of such systems namely, active and passive. Passive type patches delivery drugs instantaneously while, active patches are induced to delivery drugs. Such an active inducement may result from: (i) external manual signals in the form of light, sound, electrical current, or (ii) internal automatic stimuli in the form of enzymes, pH, temperature, analytes recognitions. One of the well-known examples for internal stimuli drug release system is glucose-responsive insulin microneedle patches. Passive drug delivery profile entirely depends on the drug formulation. A schematic representation of the types of drugs delivering smart patches, its components, materials used and fabrication methods is presented in Fig. 14. For example, chitosan exhibits slower release rate than alginates, while hyaluronic acids degrade much faster than polyesters and silk fibroins. Sustained release of drug from passive drug delivery patches were obtained by using rate-limit membranes [118]. Due to the challenges associated with active drug delivery systems in integrating electronic components for controlling drug release and offering biocompatibility with the skin, development of stimuli-response materials that offer controlled passive drug release and those that offer internal stimuli-induced drug release are investigated adequately. Such materials often fall under categories like (i) thermo-responsive particles like poly(N-isopropylacrylamide) (NIPAM); (ii) photosensitiser-loaded particles like lanthanum hexaboride (LaB6) nanoparticles, NIPAM-VP with magnetic NPs; (iii) electrically activated compounds like m-SiO2 particles; (iv) mechanical force responsive particles like drug-loaded poly(lactic-co-glycolic acid) (PLGA) nanoparticles dispersed in alginate microgels, or (v) microneedles [119].

Microneedles (MNs) are the most preferred routes for delivering drugs by DDS as compared to the iontophoresis, sonophoresis, and electrophoresis methods. The reason lies in their painless nature as MNs penetrate only the vigorous stratum corneum and epidermis, and do not reach nerve ending and blood vessels. Microneedles are conventionally classified into four categories as (i) solid MNs, (ii) coated MNs with water soluble formulations, (iii) dissolvable MNs, (iv) hollow MNs. Recently, functionalised MNs capable of responding to various signals and analytes were developed with advanced materials. Such MNs were capable of providing diagnosis, monitoring and therapeutic solutions together. Materials like hyaluronic acid, sucrose, hydroxyethyl starch 70000, poly vinyl alcohol, polyimide, silicone and ceramics are used for MN fabrication. While methods like X-ray photolithography, lithographic moulding, micro-moulding, ultrasonic welding, micromachining, ion etching, 3D-printing are used for MN fabrication [120]. Recent day smart drug delivery systems are equipped with sensors to monitor biomarkers, electronics to determine the health status; trigger DDSs to deliver appropriate drug in desired quantity [121]. Such fully integrated DDSs were developed for treating diabetes, infected wounds, epilepsy, Parkinson’s disease and ankle injury [119].

Advancements and discovery of nano-materials enabled the development of smart materials with biosensing, self-powered light-weight devices with controlled drug release, capabilities [122]. Especially, developments in stimuli-response materials and flexible electronics enabled emergence of self-sustained devices along with required biocompatibility. For instance, nano-materials releasing contact lenses are emerging as promising drug delivery systems for glaucoma treatment. In this context, polymeric materials, lipid-laden contact lenses and magnetic NP-loaded contact lenses are noteworthy [123]. Similarly, a smart drug delivery patch was developed using jute, carbon dots dispersion in cotton fabric. The patch when loaded with natural drug like, neem extract exhibited a pH-responsive drug delivery. Drug release at pH 5 was higher compared to that at pH 7 which demonstrated the patch’s pH-responsive drug release behaviour [124].

Interestingly, a combination of fused deposition modelling (FDM)-printing and stamping moulding was used to construct a thermos-responsive 4D structure which is capable of transforming to desired shape based on temperature change. The patch was demonstrated to change shape with temperature and promote proliferation of human-induced pluripotent stem cell-derived cardiomyocytes (hiPSC-CMs). The smart 4D patch is a reliable smart cardiac construct which functions as both minimally invasive cell vehicles and patches for repairing damaged myocardial tissue [125]. Similarly, a composite patch fabricated from poly(glycerol sebacate) (PGS), collagen type I, polypyrrole loaded with small molecule (3i-1000) was demonstrated to promote attachment of cardiac myoblast cells. With polypyrrole-induced cell signalling, and 3i-1000 induced cell proliferation, the composite patch could be reliably used for myocardial infarction treatment [126]. Drug delivery platforms developed from expandable polymeric material were demonstrated to deliver drug on-demand based on measured temperature changes [127].

### Wound care

**Fig. 15:**
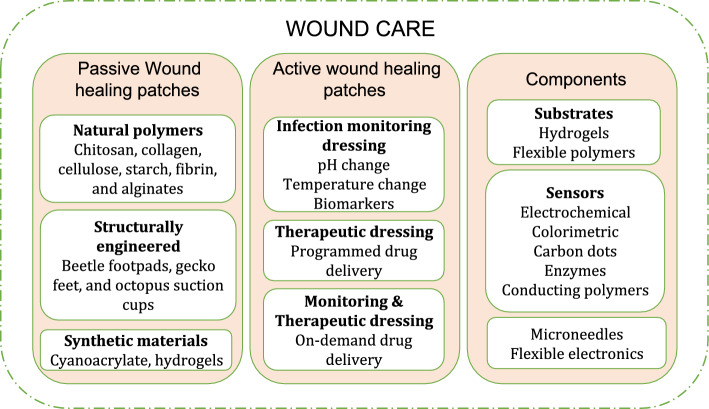
Components and functions of a smart wound care patch

Wound care appears at the intersection of non-invasive monitoring and drug delivery. Wound is any disruption or damage to living tissue resulting from accidental injury or created by surgery. Based on the healing nature wounds are of two types namely acute and chronic wounds. Chronic wounds are those that fail to heal within one month and handling such wounds is a myriad task. The primary factors that hurdle healing of wound are ischaemia (poor blood flow) and hypoxia (poor oxygenation) which lead to bacterial biofilm formation, prolonged inflammations, and cellular dysfunctions. Further, chronic wounds are often prevalent in persons with diabetic, obesity, and auto-immune diseases. Similarly, wounds affected by constant pressure, friction and swelling take prolonged duration to heal [128]. The use of natural polymer-based wound dressing materials has demonstrated wound care potential in the case of chronic wounds. Natural polymers have good biocompatibility, biodegradability and capable of maintaining a moist wound environment that promotes healing. These materials could provide comfortable long-time wearability inherent to their biocompatibility. In addition, functionalisation of such materials with phytochemicals enabled enhanced wound healing potentials in the case of chronic wounds [129]. Further, multi-functional wound dressing in the form of electrospun nanofibers and bio-composite scaffolds demonstrate excellent wound healing potentials with sustained drug release capabilities [130]. However, the emergence of wearable smart healthcare devices has created a paradigm shift in wound care and management [131]. Smart patch devices use built-in sensors and stimuli-response materials for interacting and understanding the wound environment and thus facilitate accelerated wound healing [132]. These devices find application in wound care in the form of simple monitoring devices or as both monitoring and therapeutic devices [133]. Intelligent controls aid in precise drug release at the wound site in two ways which include: (i) programmed release of drugs and (ii) on-demand release based on monitored parameters [134]. The monitored parameters for on-demand drug release are categorised into two types based on the nature of stimuli given. And they are (i) endogenic stimuli which are acquired from the wound site like pH, temperature, glucose, bacteria, enzyme and (ii) exogenic stimuli which are given by external sources like light, magnetic, electrical and thermal[135]. The functions and components that constitute a smart wound care patch are shown in Fig. 15.

In addition to electronic sensor, control-based smart wound healing patches, smart biomaterial loaded wound healing passive patches are gaining significance in wound care. These patches include: (i) natural materials like chitosan, collagen, cellulose, starch, fibrin, and alginates; (ii) structurally engineered patches like beetle footpads, gecko feet, and octopus suction cups; (iii) synthetic materials like cyanoacrylate, hydrogels. Such materials promote wound healing by offering greater adhesion at the wound site by various mechanisms like mechanical crosslinking or interlocking, and dual-networking [136]. For instance, hydrophilic polymers especially hydrogels are emerging as potential wound care materials facilitating moist environment for wounds, absorbing exudates, preventing infection and supporting tissue regeneration. Functionalised hydrogels provide enhanced wound care solutions by facilitating on-demand drug delivery by monitoring wound parameters [137]. For example, wound temperature and pH are reliable indicators for analysing wound status. Healthy skin has a pH between 4 and 6 while infections result in increasing the pH value and thus by measuring pH in wound environment, therapeutic intervention could be made. For instance, pH-responsive hydrogel was developed and demonstrated to be an effective wound dressing material indicating infection by colour change. Phenol red molecules were modified with methacrylate and copolymerised with alginate/polyacrylamide (PAAm) hydrogel matrix in order to avoid dye leaking. The patch with its colorimetric indication of pH, is a reliable substrate for printed electronics [138]. TSP gel formed from tamarind seed powder with dHAM placed on top of immature coconut fibre sutures in Wister rats demonstrated faster wound healing than control group. This example showcases the potentials of natural materials in wound healing and research progresses along nature-derived material studies [139].

However, integration of electronic sensors and control along with these biomaterials like cellulose resulted in an active and intelligent closed loop wound monitoring and drug delivery [136]. For instance, a wound pH, moisture monitoring patch was developed by inkjet printing of single wall carbon nanotube on PDMS substrate. Results demonstrated that the pH sensor achieved a sensitivity of 7.1 Ω/pH [140]. In addition, a wound healing patch capable of monitoring wound site pH, glucose, infection and mechanical deformation was developed using a grooved polyvinyl alcohol (PVA) substrate. Hydrogel fibres loaded with phenol red, glucose oxidase (GOx), carbon nanotubes and horseradish peroxidase (HRP) enzyme were installed in the grooves in PVA substrate in order to provide a colorimetric indication of the monitored parameters. In addition, the patch was demonstrated to provide photo-thermal therapy for wound healing [141]. These types of colorimetric reading were decoded by AI algorithms for making predictions on wound infection and their battery-free operation often results in the required miniaturisation [142]. Further, smart wound care patches with drug delivery capabilities are often equipped with microneedles for painless drug delivery to the wound sight. These microneedles are designed either based on biomimetic design, electroconductive design or environment responsive design [143].

Recent researches demonstrate that introduction of nerve growth factors (NGF) stimulate growth and differentiation of neurons [144]. For instance, a passive smart dressing that aids in angiogenesis by on-demand release of Co2 + with fluctuations in pH as well as neural regeneration by CXCL12, ligustroflavone, and ginsenoside Rg1 release in a sustained manner was developed by functionalising a cross-linked hydrogel. Experimental results demonstrate that the patch achieved angiogenesis and neural regeneration within 17 days [145].

### Electrical stimulations

Smart patch type devices capable of delivering precise quantity of electrical stimulations (ES) find applications in clinical procedures [146]. Application of electrical stimulations enable nerve regeneration and thus, possess potential for neural repair applications. The use of ES for nerve regeneration was first investigated by Hoffman in the year. The study demonstrated improved sprouting in denervated muscles when 50 to 100 Hz ES is applied directly on L5 spinal nerve root for 10 to 60 min. There are numerous studies that revealed nerve regeneration potential of ES therapy [147]. Stimulations both in electrical and mechanical forms are largely investigated in the field of limb prosthetics [148], artificial smart skin [149], flexible interfaces [150]. For example, an sEMG sensor for amputees wearing socket was developed employing PDMS and Silbione substrates was demonstrated to control robotic leg. The device exhibited an SNR of 24.68 dB, sensitivity of 5.17 µV N^−1^ for muscle contraction exercise which was better in comparison to an available sEMG sensor [151].

In addition, conductive scaffolds were demonstrated to promote neural regeneration. For instance, a scaffold was made by combining poly(3,4-ethylenedioxythiophene) (PEDOT)-reduced graphene oxide (rGO) hybrid microfiber with a triboelectric nanogenerator (TENG). The scaffold generates pulsed electrical signals with the help of TENG which resulted in accelerated nerve regeneration [152]. But, direct application of electrical stimulations on wounds does not show effective healing effects due to absence of antibacterial agents. However, combining electrical stimulations from triboelectric nanogenerator with antibacterials drugs in microneedle patches promoted wound healing [153]. For instance, skin wound accompanied by nerve destruction often leads to loss of sensation and unsatisfactory nerve regeneration. A self-powered smart patch (PRG-G-C) capable of providing chemokine and programmed biological-electrical cues with the help of flexible piezoelectric generator was demonstrated to accelerate nerve regeneration. Results prove that the application of such a smart patch enabled sensory restoration within 23 days [154]. Further, flexible smart wound patches capable of monitoring wound for bacterial infection, communicating wound status to smart phone and delivering electrical cues with the help of smart phone signals was developed. The smart patch utilised a DNA hydrogel-coated gate organic transistor for bacterial infection monitoring and two Ag electrodes with low contact impedance for delivering electrical cues. Such a smart patch demonstrated a highest selectivity of DNase (0.1 U mL^−1^), for 10 µM of lactic acid, 300 µM of uric acid, 5 mM of glucose, 15 mM of bovine serum albumin in bacterial monitoring experiments. And 300 µA direct current stimulation from the device was demonstrated to inhibit bacterial growth [155]. In another experiment, a 3D porous matrix loaded with chemokines and anti-senescence drugs, was demonstrated to promote wound healing and nerve regeneration by stimulating electrical conductivity [156]. A smart patch with thrombin-derived c-terminal peptide-25 (TCP-25) loaded chondroitin 4-sulfate, hydrogel patch, a pair of electrodes for electrical stimulation and an array of six biosensors for monitoring multiple wound biomarkers was fabricated and demonstrated to promote wound healing and nerve regeneration. Such smart wound monitoring device demonstrated a sensitivity of 59.7 mV while sensing wound biomarkers. This device was equipped with an electrical stimulation pulse voltage of 1 V at 50 Hz for 0.01 s during each cycle and substantial elimination of bacterial growth is registered after 3 days [157].

## Technologies that serve smart patches

Two key technologies that drive advancements in smart patch developments for the healthcare industry include: (i) advanced materials and (ii) intelligent computing and communication.

### Advanced materials

Advancements in nanotechnology and material discovery remains the key determinants driving developments in realisation of miniaturised smart sensors with storage/memorising capabilities, wireless transmission ability, as well as self-powering capabilities. Such smart materials mimic natural biological counterparts and often follow a nature-inspired design to offer biocompatible solutions. However, smart material research is still in infancy, and demands lot of research. Product realisation and commercialisation of such smart materials involve adequate testing and ethical clearance, which is often a time-consuming process.

*Biosensors.* Sensors are the primary components in a wearable healthcare device and break through advancements in sensor technologies serves as the backbone for smart patch developments. Flexible, soft and stretchable wearable sensors enable continuous monitoring of healthcare parameters in an unobtrusive way. Such sensors offer measurement in a non-invasive way by measuring analytes from various body fluids like sweat, tear and ISF [158]. Biosensors are highly sensitive and selective tools that aid in rapid diagnosis. In addition to analyte sensing biosensors could reliably detect cellular proliferation, differentiation, metabolism detection as well as stimulation responses [159]. These sensors are classified into different categories based on the sensing technique used, biofluid sensed, target analyte, type of wearable developed, type of substrate, etc., Based on the sensing technique used, biosensors are classified as physical and chemical sensors. Physical sensors measure temperatures, humidity, bio-electrical signals, pressure, shear, strain, heart rate, while chemical sensors measure a particular analyte in biofluids [160, 161]. In addition to wearable sensors, even ingestible and implantable sensors are used for continuous health monitoring [162].

Biosensors contain a biological sensing element in contact with a transducer. The sensing element often includes enzymes, antibodies, cells or nucleic acids which while coming in contact with the analyte of interest induces a biorecognition event producing a bio-signal. Strength of this bio-signal is in direct proportion to the concentration of the analyte and is detected by an appropriate transducer [163, 164]. Nanoscale dimensions of such sensors enable ability to probe even single molecules for detection and thus fast and simple detection of analytes is possible [165]. The analytes being sensed include proteins, DNAs, ions, metabolites and transduction based on optical, electrochemical and piezoelectric principles are most frequently used [166, 167]. Multiplexed sensing utilising integrated multiple sensors offer measurement of multiple analytes with a single device and at a single instance. Such devices are helpful in enabling easy identification of viral mutations [168]. Microfluidic biosensing systems in the form of chips offer such multiplexed analysis in spite of using only nano litres of the analyte [169].

**Fig. 16:**
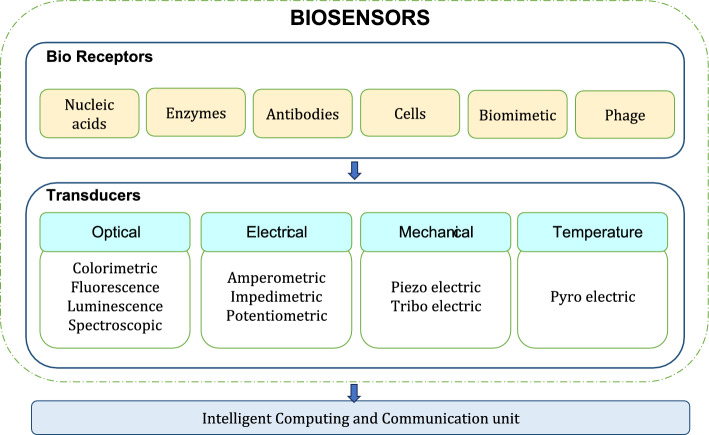
Components of a biosensor and its transduction principle

A schematic representation of types of nano-biosensors and transduction principle is presented below in Fig. 16. The key determinants that drive development of biosensor are discovery and development of advanced nano-materials, development of flexible substrates and printing technologies, each of which is discussed in below sections.

The substrates used for fabricating these biosensors determines their usability, compatibility and cost effectiveness. For instance, paper-based biosensors are emerging as potential candidates for economical disposable sensing devices for non-invasive diagnosis of diseases from bodily fluids like saliva, tear and sweat. Paper-based biosensing units take three different forms like dip-stick assays, microfluidic devices and lateral flow assays. Microfluidic devices use filter papers made from cellulose as substrates while lateral assays use nitrocellulose membrane to immobilise protein by streptavidin–biotin interactions. These sensors work on colorimetric, luminescent, electrochemical and multi-modal sensing principles [170]. Glucose biosensor was fabricated by encapsulating glucose oxidase and AIE-type gold nanoclusters into acid-sensitive zeolite imidazolate framework (ZIF)-8 nanocrystals. This nanoprobe when integrated into paper substrate, the biosensor was able to detect glucose with smartphone help [171].

Significant progress in the field of wearable biosensor patches is driven by the introduction of nano-materials (NMs) [74]. Developments in electrochemical biosensor technologies powered by nano-materials enabled effective detection of biomarkers from various body fluids like sweat, tear, saliva, interstitial fluid of skin (ISF) [1]. NMs frequently used for biosensing applications include: (i) nanoparticles that have higher carrier capacity and stability; (ii) nanowires that have higher sensitivity; (iii) carbon nanotubes that have higher electrical and thermal conductivities and larger surface area; (iv) quantum dots which are colour tuneable [172]. For instance, sensors in the form of tattoos were highly flexible and easily adhere to the skin. They present promising avenues for the realisation of healthcare monitoring devices. Such e-tattoos are developed over glossy paper and liquid bandage and are transferred to skin. Materials frequently used for realisation of such e-tattoos are silver nanowires, gold nanowires, dynamic ionic liquids, graphene inks, silk protein with carbon nanotubes, and PEDOT:PSS and these materials are printed or deposited on to flexible substrates. These tattoos were used for realising electrochemical sensors, ion-selective membranes, organic electrochemical transistors, temperature sensors and enzyme sensors [173].

Printing techniques enable realisation of miniaturised, high resolution sensors and their integration with electronic and microfluidic systems. Printing techniques like inkjet printing, screen printing, aerosol-jet printing could achieve resolutions as small as of 15 μm, 10 μm, 30 μm, respectively [174, 175]. A conductive ink was synthesized by decorating silver NPs over multiwalled-CNTs with PEDOT:PSS as binding agent by a wet chemical process. The ink traced with 9.5 μm thickness exhibited a conductivity of 28.99 S/cm and presented good adhesion over mylar and Kapton materials. The sensor demonstrated good cycle stability of 10,000 cycles and maximum stretchability of 23%. This demonstrates the potentials of a conducting ink for printed electronics [176].

*Electrochemical sensors.* Electrochemical immune-sensors are affinity based biosensors widely used for biomedical sensing applications and their performance depends on the materials, reagents used for their construction and signalling [177]. Electrochemical sensors in the form of organic transistors offer reliable analyte sensing from biofluids. Such transistors based biochemical sensors are low-cost devices with excellent mechanical, electrical characteristics which enable robust and good signal amplification capabilities [178]. Further, flexible reconfigurable biosensing devices, are realised from soft materials like liquid metals and printable inks. For example, gallium-based liquid metals are conductive as well as soft materials with low toxicity and good deformability. Such properties enable their use in the development of soft, reconfigurable bio-devices [179]. An electrochemical glucose oxidase sensor was realised by screen printing carbon electrodes and potentiostat for amperometry detection. The glucose sensor works along with a dialysis membrane and 3D microbioreactor for achieving controlled bioprocesses [180]. In certain case, transduction is enabled by NMs. For instance, dendrimers are soft polymeric NMs used widely as transduction elements in biosensors. Dendrimers like PAMAM and PPI are compatible with proteins like antibodies and thus, they can be combined with NMs and polymers for reliable biosensing applications [181]. In addition to dendrimers, peptides are powerful biorecognition elements for the development of impedimetric biosensors capable of sensing bacteria [182].

*Optical sensors.* Optical biosensing offers numerous benefits like accelerated analysis, higher sensitivity, and specificity over traditional diagnosis methods [183]. Biosensors incorporating photonic crystals (PCs) present promising potentials for use with wearable devices. Especially, PC-based sensors offer multi-target detection capabilities making them suitable for multiplexed and microfluidic environments [184]. In addition, fluorescent biosensors were recommended to be used for studying the functions of neurotransmitters and neuromodulators [185]. Further, optical sensors based on nonlinear absorption, surface plasmon resonance (SPR) and Raman dispersion are capable of sensing multiphoton effects and thus, produce output with improved sensitivity [186]. For example, fibre Bragg grating (FBG) sensor that uses an optical fibre encapsulated in fabric liner was fabricated and demonstrated to measure heart rate and respiratory rate by converting chest deformations into an optical signal [187].

*Electrical sensors.* Electrical sensors are normally categorised into three types namely, amperometry, voltammetry and potentiometry sensors. These sensors are realised on flexible substrates with printable conductive inks in different shapes based on requirement. For example, a ring-shaped capacitive sensor was fabricated by stencil printing technology. Electrode were made of printable polydimethylsiloxane (PDMS) filled liquid metals and porous PDMS acted as the dielectric. This sensor exhibited a capacitance change of − 38.7% which is 3–5 times more than existing system [188]. In another experiment, a leaf-like electrode skeleton was fabricated by physical vapour deposition of Au by sputtering and Ag nanowires are drop-casted onto the leaf skeleton. The fabricated porous structure displayed a uniform sheet resistance of 5 1ΩSq-1 and demonstrated to be a potential candidate for measuring bio-signals [189]. These types of sensors could be reliably tailored for various functions by introducing materials with varied permittivity or by adapting unique design patterns. For instance, dielectric polymers with specific permittivity could be a reliable material for use in capacitor, energy storage and flexible electronic devices. Dielectric polymers exhibit high permittivity, excellent dielectric properties and flexibility. The permittivity of such materials depends on the type of polarization, which includes interfacial polarisation (IFP), relaxation ionic polarisation (RIP), orientational polarisation (OP) in the relaxation regime (slower) and electronic polarisation (EP) and the instant ionic polarisation (IIP) in the resonance regime (faster) [190].

*Mechano-electric sensors.* Sensors capable of converting mechanical energy to electrical energy fall under this category. Once realised as huge electromechanical energy conversion systems could realise as smart materials with the advent of nanotechnology and MEMS design. These piezoelectric and triboelectric devices when integrated to wearable devices could function as simple sensors or as nanogenerators. For instance, triboelectric nanogenerators that could harvest energy from human body motions are integrated with smart patches and were demonstrated to power the device functions [12]. Economical and simple fabrication of such devices with multiple functions were possible with recent printing technologies. Printing functional nano-materials over polymeric substrates emerged as a reliable fabrication method for realising self-powered temperature sensing patches [32]. Further, self-powered sweat sensing patches were developed utilising piezoelectric ceramic material for converting mechanical energy generated during exercise into electrical energy and a hydrophobic and hydrophilic microchannel system for sweat collection and near-field communication (NFC) is used to transmit sensed data to mobile and cloud [45, 48]. Similarly, a self-powered smart patch capable of diagnosing cystic fibrosis by measuring sweat conductivity instead of conventional sweat chloride level was developed. This device was powered by a paper battery that gets activated when fluid to be measured was added to it. The output of the device is fully dependent on the conductivity of the liquid sample and demonstrated a sensitivity of 95% and specificity of 100% [191]. Even converting temperature to electrical energy based on pyroelectric principles is realised with material advancements [192].

Biosensors when equipped with point-of-care diagnostic features could provide instantaneous results. For instance, colorimetric patches could readily provide qualitative diagnosis results and patches integrated with embedded processors could provide PoC diagnosis. However, a wireless transmission and integration with a smart device enables remote access capabilities. Even wireless transmission capabilities can be realised from smart sensing materials. For instance, temperature and motion sensing smart patches were equipped with wireless transmission abilities based on triboelectric and electrostatic induction principles. A fabric electrode developed using carbon nanotubes (CNTs) on fabric and silver nanowires coated on PDMS were used for receiving wirelessly transmitted signals from the patch sensor. The transmission achieved an efficiency of 26.6% for a receiver distance of 1 cm. Thus, presenting a convenient and versatile solutions [193]. Though materials with wireless transmission abilities, power generation abilities are found their use for powering and wireless communication are still in infancy and thus, most present-day smart devices use available methods for realising wireless transmission and powering requirements. Wireless communication technologies like Bluetooth, Zigbee, infrared, radio frequency identification (RFID), Wi-Fi and near-field communication (NFC) technologies aid in connecting wearables to smart devices [194]. These topics that enable communication and computing of sensed data are covered in the next section.

### Intelligent computing and communication

Intelligent computing section includes recent advancements in computing techniques that enable communication protocols, information/data handling and transfer, cloud-based storage and computing, artificial intelligence models. These technologies provide remote monitoring and healthcare solutions integrated with the smart patch devices thus, serving the healthcare industry in numerous ways. Specifically, Internet of Things (IoT) plays major role in providing remote access while artificial intelligence techniques provide the necessary prognosis.

#### Internet of Things—cloud storage/computing

**Fig. 17:**
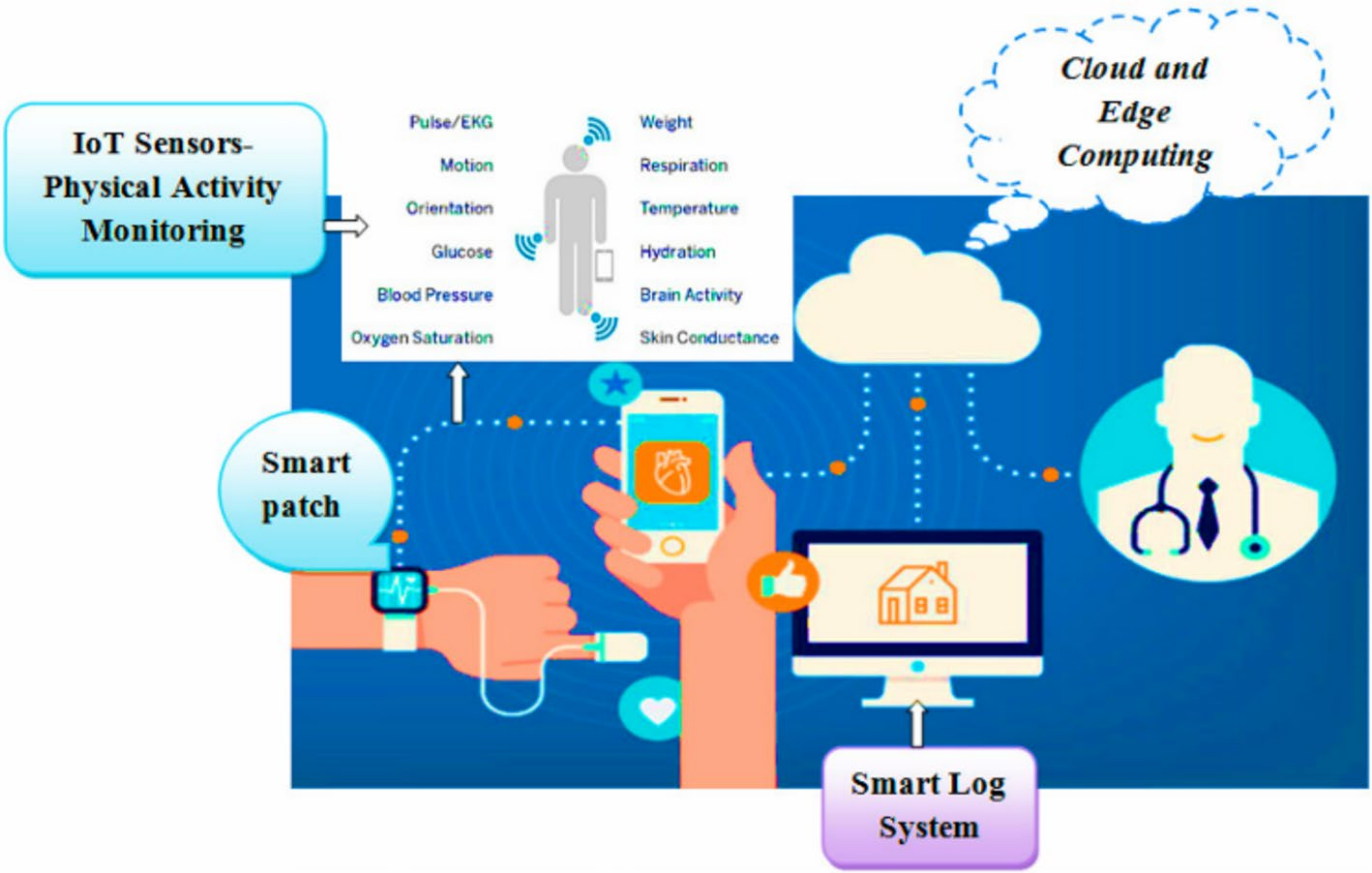
Schematic concept of IoT-enabled wearable smart log patch. Reproduced with permission from [200] under the terms of Creative Commons Attribution (CC BY) license (http://creativecommons.org/licenses/by/4.0/)

Internet of Things (IoT) is revolutionising the healthcare industry by facilitating diagnostic and therapeutic solution at the users door step. IoT helps healthcare provides to extend their support to patients even outside the hospital walls [195, 196]. It helps patients adhere to treatment plans and enable caretakers to take proactive actions. In addition, it enables finding exact location of patients and elders and extend timely help [197]. As per WHO, IoT healthcare users are of four groups namely, (i) patients or general public for self-monitoring; (ii) healthcare providers; (iii) public health managers; (iv) data services. Five main components that drive IoT-driven remote healthcare solutions include: (i) sensors that monitor bio-signals; (ii) short-range communication networks that enable signals from sensors to reach a smart device; (iii) middleware that ensure the data collected from the sensors reach the cloud server; (iv) cloud computing that enables data storage, processing, analysing and alerting functions; and (v) IoT application that provides the user interface [194]. For instance, an end-to-end patient monitoring system for COVID-19 detection and monitoring was developed following a 3-layer architecture which includes: (i) data acquisition layer that has wireless sensor nodes; (ii) cloud storage and fog computing; and (iii) user interface layer which is usually a mobile application [198]. A flexible biosensor patch capable of recording ECG, BP, temperature, along with GPS location of the person and wirelessly transmitting them to mobile by BLE module was developed and demonstrated to acquire real-time data as well as enable cloud-based storage for analytics. The device uses 1D CNN for BP estimation and the estimates were conformed to adhere to medical standards [199]. Smart log patch device capable of collecting multiple physiological signals and processing them using Edge computing—Bayesian Deep Learning algorithm was developed and demonstrated to aid in real-time monitoring and producing prognostic results with the help of IoT technologies [200]. A schematic of IoT-based smart healthcare monitoring system is given in Fig. 17.

#### Artificial intelligence (AI)

Artificial intelligence techniques are a boon for medical diagnostics enabling automatic detection of diseases. Specifically, AI techniques enable quick, automated diagnosis/predictions feasible from high dimensional/molecular data obtained from biosensors in smart patches. With the inherent power of carefully developed AI tools, challenges that exist in learning features from small data obtained from electrochemical biosensors were rectified. By combining, multiple measurements of biomarkers, generative models, cumulative predictions of different models, AI tools provide improved performance in comparison to manual predictions [186, 201]. AI techniques involve post-processing of biosensor output which are primarily used for providing (i) diagnosis of sensed data; (ii) remote access to sensed data; (iii) handling multi-modal data and providing quantitative results; (iv) providing user interface for easing analysis; (v) providing feedback-based control in the context of drug delivery and warning systems; (vi) storage of analytics. AI inclusion could provide a fully integrated system that could provide enhanced healthcare solutions. Both the AI techniques namely, machine learning (ML) and deep learning (DL) are capable of providing reliable biomedical predictions. However, ML algorithms demand, a feature extractor to learn features from unstructured data while DL methods are capable of extracting the features by themselves.

Machine learning-based pipeline often includes data cleaning, data-preprocessing, feature extraction, feature reduction, detection, predictions, classification tasks [202]. Machine learning algorithms commonly used for analysis are classified into two major groups namely, (i) non-neural algorithms and (ii) neural algorithms. Non-neural algorithms usually include principal component analysis (PCA), support vector machines (SVMs), hierarchical cluster analysis (HCA), decision trees (DTs) and random forest (RF) algorithms while neural network-based algorithms include feedforward neural networks (FNN), recurrent neural network (RNN), convolution neural network (CNN) [203]. For instance, a machine learning (ML) pipeline was developed for near real-time spectral analysis of saliva samples for accurate identification of SARs-CoV-2. A hyperspectral hardware platform which includes two spectrometers, a tungsten halogen light source and a cuvette holder was used for study. Samples loaded in cuvettes and their spectra are measured with the two spectrometers and the resulting signals are concatenated into a single feature vector and then fed as inputs to ML model. Random forest classifier, XGBoost ensemble method, H2O AutoML framework and pattern discovery engine (PDE) were used for making predictions and results demonstrate all the four methods could achieve high specificity and sensitivity in the range (0.89 to 1) and (0.74 to 1.0) [204]. The introduction of computer-aided design tools and computational methods enabled faster drug discovery, development of molecular dynamics simulation, virtual screening, molecular docking and hybrid methodologies [205, 206]. ML-assisted prediction and classification tasks have enabled the realisation of faster, low-cost point-of-care devices for diagnosis [207]. Introducing embedded controllers for executing AI algorithms on-device minimises transmission delays and execution delays. Such a design can be efficiently realised by having detection, prediction and feature extraction part of algorithm on device while executing classification tasks in computers or smart phones [117].

DL methods are capable of processing both unstructured data as well as structured data. In the context of smart patches, images obtained from colorimetric patches, numerical data based on biochemical sensor outputs and time series data based on bioelectric signal sensors are all processed by DL algorithms. Considering image inputs, preprocessing stage normally includes filtering, image resizing, and rescaling for improved feature extraction and predictions. However, in case of bio-electrical signals, the obtained signals are filtered, amplified and often converted to digital signals for processing. CNNs are efficient in processing image inputs while RNNs, LSTMs and transformers are efficient in making time series predictions. The primary advantage of DL methods is their ability to make predictions based on their acquired knowledge which is significant in the case of clinical decision-making. For example, colorimetric reading of analyte concentration is often affected by biofluid pH and lighting conditions. Images of colorimetric changes obtained after sensing tear biomarker are retrieved using smartphone camera and the corresponding RGB values are obtained using OpenCV library in Python. Analyte concentration prediction is carried out using six DL architectures namely, three-channel convolutional recurrent neural network (3D CNN-GRU), the three-channel convolutional neural network (3D-CNN), the three-channel recurrent neural network (3D-GRU), the single-channel convolutional recurrent neural network (1D-CNN-GRU), the single-channel convolutional neural network (1D-CNN), and the single-channel recurrent neural network (1D-GRU). Experimental results revealed that 3D-CNN-GRU exhibited minimal loss in predicting concentration of Ca2 + , vitamin C, proteins. A cloud server data analysis system (CSDAS) was developed integrating multi-channel CNN-GRU model [57]. Further, AI tools enable enhanced wound care solutions by their inherent capability to analyse huge sensor data, extract meaningful insights about wound healing status [137]. Inherent to the importance given to early detection and control of TB after the pandemic, a framework for research and development of immune profiling and immune biomarker studies was suggested in literatures [208]. Similarly, ResNet-50 pre-trained on ImageNet dataset is used to classify chest X-ray images to classify them into COVID-19 and normal cases [198].

In the context of physiological signal processing, AI tools enable efficient emotion recognition based on emotion relevant features of various physiological signals like EEG, ECG, EMG, EOG, heart rate, galvanic skin response GSR [209]. For instance, heart rate variability from ECG was used to classify stress level in automobile drivers. And experimental results demonstrated that multiple linear regression (MLR) algorithm is able to classify stress levels with 90.32% accuracy [210]. Even blood pressure measurements are reliable biomarkers in determining stress [211]. Further, multi-modal signal obtained from PPG, GSR and skin temperature are used for emotion recognition with SVM, KNN and DT algorithms. And results supported the efficacy of using multi-modal signals as compared to single mode signals [212]. In addition to stress monitoring, gait analysis and fall detections could be achieved efficiently with AI tools [213]. AI algorithms together with electrochemical sensing plays a major role in predicting glucose from non-invasive measurements [214]. Similarly, artificial intelligence techniques were demonstrated to make accurate seizure prediction, by learning patient-specific seizure occurrence periods using AI algorithms [215]. Further, in the context of wound status monitoring, AI powered smart patches were demonstrated to offer on-demand drug delivery based on wound pH [131].

## End-users, challenges, opportunities

### End-user benefited from smart patches

#### Physician–patient interaction

Online Health Communities (OHCs) have played a major role in providing healthcare services in pandemic situations by reducing hospital congestions and relieving resource shortage by improving utilisation of available resources. An important challenge that needs to be addressed for the successful implementation and running of OHCs are the physician–patient communication and patient compliance to treatment. Physician–patient communication has been identified as a critical factor in patient satisfaction and compliance [216–218]. Effective communication not only influences patient satisfaction, but also impacts patient engagement and trust in physicians, ultimately leading to improved health outcomes [219–221]. Furthermore, the use of electronic communication tools has been proposed to bridge the gap between acute care and primary care physicians, emphasising the importance of seamless information exchange in healthcare delivery [222]. The use of telemedicine and telemonitoring technologies has been recognised as a means to enhance remote healthcare delivery, especially in the context of the COVID-19 pandemic. These technologies have the potential to improve patient access to healthcare services and enable real-time interaction between patients and physicians, addressing the challenges of information transfer in medical consultations [198, 223, 224]. Quantifying the information exchange quality by some means and using it as feedback could help improve treatment quality and patient satisfaction [225].

Smart patches have emerged as a promising technology to bridge the gap between patients and physicians, enabling a closed-loop diagnostic and therapy systems with feedback-based control. Such systems are capable of changing the healthcare paradigm to become a patient centric system as against the present-day hospital centric system [226]. Smart patches are integrated with advanced technologies, such as artificial intelligence and cloud computing to enable remote monitoring and early detection of health conditions [227].

### Rehabilitation care

Wearable healthcare monitoring devices have the capabilities to fill the shortcomings in healthcare services provided by healthcare providers. Especially, for senior citizens and patients with disabilities under rehabilitation [228]. IoT enabled wearable devices could: (i) provide simple record keeping and paper work; (ii) avoid manual errors; (ii) provide a cost-effective solution; (iv) eliminates limitation with distance; (v) early detection of chronic diseases; (vi) improved medical management [229].

Smart patch wearable devices are a boon for monitoring people at risk. This category includes people who suffer from (i) congestive heart failure; (ii) chronic obstructive pulmonary disease; (iii) sleep apnoea; (iv) dementia; (v) age-related disabilities. Healthcare costs associated with this community is higher and they often demand a caretaker (often family members) for leading a good quality of life [195]. In addition to this, people who are under rehabilitation therapy due to (i) post-operative recovery, (ii) disabilities, (iii) special abilities demand a trained care taker for discharging healthcare services [162]. Smart patch devices could offer the required services by (i) monitoring physiological parameters; (ii) early detection of critical health changes; (iii) alerting caretakers to take actions, and providing tailored drug delivery [230].

### MSD prognosis

Work-related musculoskeletal disorder (MSD) could be diagnosed early with the help of data analysis tools. Such a prognosis demands heterogeneous data pertaining to patient health data and recent day smart sensor devices are capable of providing such data from wearables and electronic health records [231]. These smart patch devices provide ambient intelligence (AMI) capabilities powered by smart biosensor, AI and IoHT [232]. These capabilities support the feasibility of using smart patches for MSD prognosis [233, 234]. As an initiation towards musculoskeletal health monitoring an IoMT device capable of grading muscle power was developed. EMG signals are received from forearm muscles are processed by node microcontroller and processed signals are transmitted to Google spreadsheet in Google drive. This data was processed by a CNN in Google Colaboratory and its decisions are sent as mail. Such a system is enabled by a CNN trained from EMG muscle power grading data [235]. Further, a flexible platform for remote MSD management and sensor-assisted physiotherapy was developed with the objective of enabling remote monitoring and virtual coaching to patients. Such a system (ePhysio) used inertial measurement sensors along with Bluetooth for monitoring patient movements and updating status with the rehabilitation hub. ePhysio is based on wearable textile and enables single-user physiotherapy, indoor group therapy as well as outdoor activities. However, these systems can be developed as patch devices which increases patient comfort and compatibility [236]. A survey of self-monitoring practices among MSD patients revealed that, majority of the people depend on non-digital means of monitoring however, the effectiveness of using digital health applications for MSD monitoring was supported by 78% of the participants. In addition, non-perceived nature of symptoms leveraged people to never track their MSD health [237].

### Athlete health monitoring

Sports and fitness industry has harnessed huge benefits from technological advancements and is constantly evolving with the changes. Real-time health monitoring enables a swift view of an athlete’s physiological condition which is highly significant in optimising their performance and preventing injuries by adjusting training parameters. Studies reveal that monitoring an athlete’s physical and mental health continuously aids in identifying associated difficulties early and take actions to help optimise training. Such a monitoring ensures an athlete harvests maximum potential out of training. Health monitoring develops awareness and accountability among athletes [238, 239]. These results demonstrate the importance of continuous health monitoring for athletes. However, there are limitations with the conventional athlete health monitoring devices. The primary factor being their inaccurate predictions followed by rigidness and discomfort which prevents their prolonged use [240]. However, the use of smart flexible patch devices could overcome the limitations with conventional devices.

### Challenges

A survey of determinants that decide a wearable device’s acceptance revealed that physical features, functional features, behavioural features, brand, and safety of a product top the list that customer’s investigated [241]. Technology Acceptance Model (TAM) based studies reveal that privacy concerns, health concerns and potential risk associated hinder the acceptance of IoMT devices among public [242]

#### Device safety and material safety

When it comes to introduction of new materials in biomedical monitoring devices, major challenge resides in the biocompatibility of the material to provide safe solutions. And second important factor is translation of research to clinical product as most research interventions fail clinical trials [243]. These two challenges are addressed by nature-inspired design and improving interaction between engineers and physicians.

*Nature-inspired designs.* One of the primary requirements for effective smart patch devices is ensuring the patch makes a conformal contact with the sensing surface. However, a patch when held over the skin for a longer duration may result in bacterial proliferation at the interface and thus, biocompatible adhesive materials are of high demand. Researchers have started searching for nature-inspired solution to tackle this requirement. For instance, switchable adhesion as inspired from snail was developed by utilising Ga liquid metal nanodroplet-based polymer materials (GxPP). GxPP was developed by incorporating polydimethylsiloxane (PDMS) into polydopamine (PDA)-modified Ga nanodroplets (PDA-Ga). Temperature dependent adhesion was demonstrated when Ga melts to liquid at 29.8 °C making the modulus of GxPP to reduce to ~ 50% of its normal value (22.1 kPa from 40.4 kPa). In addition, the patch showed antibacterial effect against *E. coli* and *S. aureus* [244].

*Physician–engineer interactions.* In order to facilitate faster transfer of technology from research to clinical use, researchers recommend to strengthen partnership between physicians and engineers. Such an interaction enables closed loop feedback. A clinician can provide limitations with the existing system which directly reveals opportunities for improvement. Second, physician can aid in developing disease models by providing clinical samples. Third, they can provide the necessary feedback throughout the device development phase so that the device gains usability [227].

#### Security and data privacy

**Fig. 18:**
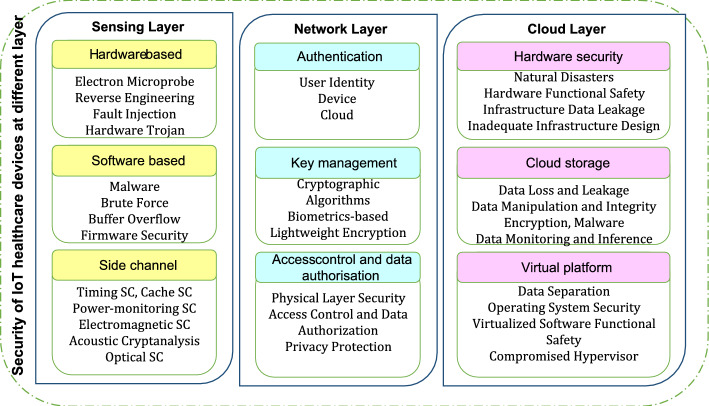
Classification of security at various level of an IoT healthcare device [247]

Acceptance and adoption of wearable technologies is greatly affected by privacy and security concerns. Secure-design frameworks that enable security from manufacturing till deployment and maintenance is recommended for easy acceptance of smart medical monitoring devices [245]. Methods to analyse security risks, such as Bayesian Attack Graph depicting likelihood of cyber-attacks are recommended for ensuring security [246]. Security an IoHT device should be addressed in three different layers namely, sensing layer, network layer and cloud layer. Security in sensing layer should include: (i) hardware based security, (ii) software based security and (iii) side channel security; whereas in the network layer, security is ensured by (i) authentication, (ii) key management, and (iii) access control and data authorisation. In the cloud layer, security is ensured by: (i) hardware security, (ii) cloud storage security and (iii) virtual platform security [247]. Classification of security requirements at different layers of an IoT healthcare device is portrayed in Fig. 18.

Introducing socket-based security in client–server architectures which are connected to mobile, cloud as well as fog nodes results in (i) difficulty to manage, (ii) demands huge processing and network costs, and (iii) occupies storage. The questions on data security, privacy related to cloud storage, remote accessing have motivated researches improve network security by introducing block-chain enabled Internet of Healthcare Things or Medical Things (IoHT or IoMT) framework for medical applications. Blockchain technology was devised as the key technology in solving security, data privacy of IoMT [248]. For instance, Blockchain enabled socket remote procedure call (RPC)–IoHT framework was demonstrated to offer the desired distributed security at reduced costs for smart medical applications [249].

#### Lack of awareness

Acceptance of smart healthcare monitoring devices is primarily ruled by three factors namely, (i) device properties like comfort and safety; (ii) data relevancy; and (iii) user-centred factors like ease of use, perceived value and social acceptance [117]. Awareness about the benefits of using smart wearable patches for continuous physiological monitoring should be created among patients. Highlighting the benefits harvested in terms of (i) early anomaly interventions; (ii) continuous support from caretakers; (iii) minimisation of treatment and monitoring cost could facilitate faster acceptance of AI-based continuous monitoring solutions [250]. Table 1 shows the summary of challenges in realisation of smart patch devices.

**Table 1:** Summary of challenges in realisation of smart patch devices

Sl. no.	Ref. no.	Challenges	Reason	Rectification options/opportunities
1	[244] [136] [134]	Smart patch adhesive	Bacterial growth at the skin, patch interface	Flexible adhesion—inspired from snail, marine mussels, octopus’ suckers and tree frogs’ toes
2	[227]	Time required for technology transfer	Difficulties in clinical trials and failed clinical trials	Physician–engineer interactions Diversifying research teams
3	[251]	Ethical regulations	Potential toxicity and health risk associated with highly sensitive nano-materials	Adapting adequate safety measures in research laboratories and getting real informed consent from research participants
4	[252]	Ethical regulations	Accountability of AI for harms if any	Establishment of ethical standards for AI use and getting consensus from stakeholders
5	[247] [245]	Adoptability of wearables	Consumers' health beliefs and privacy protection	Methods to analyse security risks, ensuring privacy protection by blockchain
6	[250]	AI Acceptance	Lack of awareness	Facilitating training and introducing interpretability to devices

### Future advances

Inherent to its infancy, smart patches hold lot of opportunities for development. Research interventions towards developing energy-efficient devices, closed loop healthcare systems, rehabilitation aids and prosthetics, secure data transfer are adequately investigated. A few of the most promising opportunities include:

*Self-powered design.* Energy harvesting methodologies such as triboelectric, thermoelectric, and biofuel cells realised with the help of novel materials are capable of generating the necessary energy from body heat, motion, or biochemical reactions in sweat or interstitial fluid. Thus, they provide the necessary power to smart patch devices. Such a self-powering mechanism enables the devices to operate without external batteries [253, 254].

*Extended biofluid sensing.* Extended sensing towards multi-analyte diagnostics aids in comprehensive assessment of immune responses, infectious diseases, and neurological conditions [255, 256].

*Closed loop drug delivery.* Drug delivery patches are heading towards enabling biomarker driven controlled drug release thus enhancing therapeutic efficacy and safety [257].

*Personalised and predictive healthcare solutions.* With the integration of AI algorithms, future smart patch devices will be capable of providing predictive analytics and disease prognosis. Further, patient-specific models that provide personalised treatments especially, for chronic conditions such as diabetes, cardiovascular diseases, and neurodegenerative disorders are feasible in near future [258].

*Ensuring data security and speed.* Incorporation of Blockchain technologies to ensure data security and leveraging the power of Edge Computing for localised data analysis for real-world deployments. [259–262].

*Collaborative research and standardising regulatory frameworks.* In spite of all these technological advancements, collaborations between academia, industry, and health authorities are highly encouraged for faster technology transfer, developing guidelines for ethical use of technology and ensuring adherence to regulatory standards [263].

This review presented the current advancements in smart patch technologies and provides a framework for researchers, clinicians, and industry stakeholders to understand the state-of-the-art and strategically advances in smart patches research. Smart patches represent an evolution in wearable medical technologies offering real-time, non-invasive, personalised health monitoring and therapeutic interventions. Powered by developments in nanoengineered materials and intelligent computing that enable flexible electronics, miniaturised and efficient biosensing platforms, predictive models, and faster communication between devices, smart patches are transforming the healthcare industry. Despite these progresses, challenges exist in the form of biocompatibility, data privacy, power efficiency, and large-scale manufacturability. However, with sustained initiative in translational research, policy development, and ethical deployment, smart patches are poised to become indispensable tools in next-generation digital and personalised healthcare ecosystems.

## Materials and methods

An exhaustive search of the literature pertaining to smart patches that include journals articles, reviews, pre-prints, conferences, book chapters is carried out. Literatures identified from their titles are shortlisted by careful reading of the abstract. Shortlisting criteria primarily emphasised on (i) smart patch types devices for use with skin, ocular, cochlear, oral and nasal microenvironment; (ii) materials that enable smart sensing with biocompatibility and biodegradability; (iii) unique fabrication methods that enable device miniaturisation as well as smart performance; (iv) technologies that facilitate smart sensing—in terms of sensing efficacy, signal processing, computing and communication; (v) challenges that need to be addressed for reliable and persistent growth of smart patch technologies. The literatures shortlisted from abstract reading are carefully analysed and relevant details were gathered and presented in readily accessible (text, diagrams and tables) form.

## Data Availability

Not applicable.
